# A Perspective on Modelling Metallic Magnetic Nanoparticles in Biomedicine: From Monometals to Nanoalloys and Ligand-Protected Particles

**DOI:** 10.3390/ma14133611

**Published:** 2021-06-28

**Authors:** Barbara Farkaš, Nora H. de Leeuw

**Affiliations:** 1 School of Chemistry, Cardiff University, Cardiff CF10 3AT, UK; FarkasB@cardiff.ac.uk; 2 School of Chemistry, University of Leeds, Leeds LS2 9JT, UK

**Keywords:** nanoparticles, density functional theory, magnetic hyperthermia, magnetic anisotropy

## Abstract

The focus of this review is on the physical and magnetic properties that are related to the efficiency of monometallic magnetic nanoparticles used in biomedical applications, such as magnetic resonance imaging (MRI) or magnetic nanoparticle hyperthermia, and how to model these by theoretical methods, where the discussion is based on the example of cobalt nanoparticles. Different simulation systems (cluster, extended slab, and nanoparticle models) are critically appraised for their efficacy in the determination of reactivity, magnetic behaviour, and ligand-induced modifications of relevant properties. Simulations of the effects of nanoscale alloying with other metallic phases are also briefly reviewed.

## 1. History of Use and Study of Metal Nanoparticles in Biomedicine

Metal nanoparticles (MNPs) have been attracting researchers’ attention for over a century, owing to the diversity in their properties and the unique phenomena that only exist at the nanoscale and have unlocked many new pathways to implement metals as technology materials. However, MNPs have been used, albeit unknowingly, long before modern ages. They were an integral part in the cosmetics of the ancient Egyptians, whereas the Romans were famous for their workmanship of stained glass which evolved from the absorption of light through gold and silver MNPs—the most renowned example being the Lycurgus cup. The trend of dyed glass and metal-resembling glazes on ceramic pottery continued deep into the Middle Ages before the origin of the colouring effects was linked to the presence of optically active colloidal nanosized metallic particles in 1857 by Michael Faraday. Today, efforts to unravel new tuning strategies leading to desired properties of MNPs continue to grow, and utilisation of such nanotechnology has moved a long way from stained glass, reaching an extensive range of confirmed and potential applications in catalysis [1,2,3,4], electronics [5,6,7], optics [8,9,10,11], information storage [12,13,14], and finally, medicine [15,16,17,18,19].

MNPs can exist as common single element structures, constituting alkali/alkaline, transition, or noble metals, or they can be composites of two or more different metals, known as nanoalloys. Their physical and chemical characteristics are determined not only by the chemical composition as in the respective bulk materials, but also by their size and morphology. Therefore, tailoring of the MNP properties depends on the strict control of each of these parameters. Only recently have advances in experimental equipment and methods led to the synthesis of MNPs of a uniform size distribution with directed morphology control of particles synthesised in solution, giving rise to the development of numerous production techniques. Today, procedures to generate MNPs with extensive control over their size and shape are well established, even to the point of reaching atomic-level precision in cluster and MNP synthesis, isolation, and deposition on a support [20,21]. Such progress has enabled the correlation between the MNP properties and size and shape effects, with multiple examples cited in the recent literature [22,23,24,25,26,27,28]. Nonetheless, MNPs are principally neither isolated nor clean, rather frequently stabilised by solvent or surfactant molecules or in form of particle aggregates, the presence of which makes the structural determination cumbersome. Consequently, experimental characterisation techniques still face significant challenges in assigning the influence of structural parameters on certain properties over the size distribution of the synthesised MNPs [29], and computational simulations have been shown to significantly contribute to the unravelling of size, morphology, and environmental effects on properties of individual MNPs [30,31,32,33,34].

In recent years, the emerging potential of MNPs with specific magnetic properties (called magnetic metal nanoparticles, mNPs, in the remaining text) has brought further advances in biomedicine. Their response to an externally applied magnetic field, in combination with easy conjugation of the metallic surfaces with various functional groups present in biomolecules, antibodies, and drugs of interest, has opened up a completely new range of biomedical applications, where magnetically induced preconcentration, identification, and separation are merged with targeted medical analytes in a single agent at the nanometre scale. This approach has allowed the development of drug delivery with reduced distribution of medical substances in untargeted tissues and improved contrasts in body scans through magnetic resonance imaging, whereas hyperthermia therapies have progressed from treatments increasing whole-body temperature to treatments with completely localised magnetically induced heat generation, as described in the following text. Initially, the focus was on the use of magnetic nanoparticles of biocompatible metallic oxides, mainly magnetite/maghemite, but upon the failed efforts to sufficiently improve their magnetic moments to fulfil the requirements of the therapies, naturally magnetic metallic counterparts, mNPs, have started to generate an appreciable amount of interest. Many of such mNP agents are already approved for clinical use, but the search for those with superior response and higher efficiencies at safer external field strengths continues, including less obvious material choices, such as Co mNPs, as promising candidates.

A comprehensive, although not by any means complete, summary of magnetic core NPs with protective coatings designed for biomedical purposes can be found in Table 1. There are many reviews on the role of iron oxide magnetic nanoparticles in biomedicine, with details behind the exhaustive efforts to adjust their properties for specific applications [35,36,37,38,39,40,41]. However, the topics of the current review are metal nanoparticles, the interplay between their physical and magnetic properties as they relate to the performance in biomedical applications, and how to approach and predict this dependence from a computational modelling perspective, focusing on the example of cobalt mNPs.

### 1.1. Biomedical Applications of mNPs

#### 1.1.1. MRI

Magnetic resonance imaging (MRI) is a powerful, noninvasive, and sensitive tomographic visualisation technique widely used in biomedicine to obtain high-resolution scans of body cross-sections. An MRI image originates from the measurement of nuclear magnetic resonance (NRM) signals that are collected as responses of abundant water protons present in biological tissues to the applied magnetic field [42,43,44]. In rare cases, signals are detected from other nuclei, such as C^13^, P^31^, or Na^25^. A strong static magnetic field is first applied to align the magnetic moments of proton nuclei, which are then deflected in the transversal plane upon the application of a short radiofrequency pulse. Magnetic moments spontaneously return to the original longitudinal direction of the magnetic field, and the time necessary for the complete realignment is called relaxation time. One can distinguish between the T1 relaxation time corresponding to the longitudinal recovery and the T2 relaxation time of the transversal decay. Both are sources of tissue contrasts in MRI scans, which depend on the net magnetic effect of a large number of nuclei within a specific voxel of tissue. Contrasting black and white areas of the MRI image correspond to the disproportionate T1 and T2 proton relaxation times of various biological tissues as a consequence of differences in their compositions and proton density, resulting in distanced signal intensities. However, the limited virtue of these differences can sometimes cause low sensitivity of the technique, resulting in inadequate image contrasts for certain clinical objectives. 

**Table 1:** Classification of magnetic NPs based on the core and coating materials and their respective applications.

Magnetic Core	Reported Coating Materials	Application	Ref
**Metal**			
Fe	polymers	MRI, drug delivery	[45,46,47]
	iron oxide	MRI, hyperthermia	[48]
	Au	drug delivery, photothermal therapy	[49]
Co	organic acids	drug delivery, hyperthermia	[50]
	polymers	MRI	[51]
	Au	MRI, gene transport, hyperthermia	[52,53,54,55]
FeCo	graphite	MRI, optical imaging	[56,57]
	CoFe_2_O_4_	hyperthermia	[58]
	Au	MRI, medical labelling	[59]
FePt	Au	MRI, photothermal therapy	[60,61]
	organic acids/thiols	biosensors, MRI, CT	[62,63,64,65,66]
	SiW_11_O_39_	hyperthermia	[67]
	polymer + SiO_2_	drug delivery	[68]
FeNi	polymers	hyperthermia	[69]
FeNiCo	propylene glycol	hyperthermia	[70]
**Oxide**			
Fe_3_O_4_	SiO_2_, TiO_2_	MRI, photokilling agents	[71,72,73]
	dextran, DMSA	MRI	[74]
	Au, Ag	MRI, immunosensor, photothermal therapy	[75,76,77,78]
Fe_2_O_3_	SiO_2_	MRI, biolabelling	[79,80]
	polymers	MRI, biolabelling, drug delivery, optical imaging	[81,82]
MnFe_2_O_4_	polymers and organic acids	MRI	[83,84]
CoFe_2_O_4_	polymers and organic acids	MRI, drug delivery, hyperthermia	[84,85,86]
	Au + PNA oligomers	biosensors, genomics	[87]
NiFe_2_O_4_	polymers and organic acids	drug delivery, hyperthermia	[84,88,89,90]
	polysaccharides	MRI	[91]
MnO	Au	MRI	[92]
	polymers and organic acids	MRI	[81]
	SiO_2_	biolabelling	[93]

**Figure 1:**
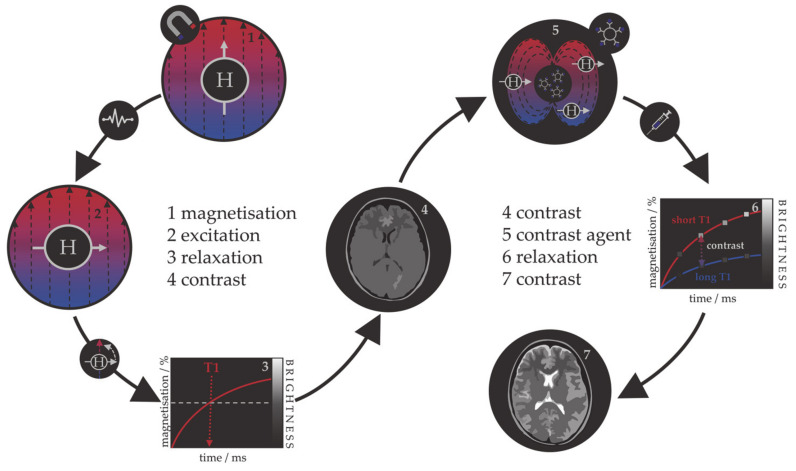
Schematic representation of the principle behind the MRI contrast agents.

Because the relaxation process involves an interaction between the protons and their immediate molecular environment, it is possible to administer MRI contrast agents that alter the magnetic characteristics within specific tissues or anatomical regions and improve the image contrast [94,95,96]. Those contrast agents are individual molecules or particles with unpaired electrons (paramagnetic metal–ligand complexes or magnetic particles) that produce inhomogeneities in the magnetic field causing a rapid dephasing of nearby protons and change in their relaxation rate. Contrast agents can be divided into those that shorten the longitudinal recovery time, resulting in a brighter image, i.e., positive or T1 agents, and those that shorten the transversal decay time, i.e., negative or T2 agents. The principle of MRI and the use of contrast agents is shown in Figure 1. Contrast agents are used in 40–50% of all MRI examinations.

The first paramagnetic complex approved in 1987 for use in cancer patients to detect brain tumours was gadolinium(III) diethylenetetraamine pentaacetic acid (GdDTPA) [97]. With rising concerns over the safety of Gd complexes, which have been found to remain in the body after multiple MRI scans, the World Health Organization (WHO) issued a series of restrictions on their use as contrast agents in 2009 [98,99]. This stimulated intense interest in creating responsive superparamagnetic T2 agents that show higher biocompatibility and safety. Currently, the majority of T2 contrast agents are iron oxide based superparamagnetic NPs (SPIONs) coated with dextran, silicates, or other polymers with variable T2 relaxivities [100,101,102]. Recent studies investigating the transformation of SPIONs into T1 contrast agents have generated some promising results, but effective contrast enhancement is still lacking, due to the unknown relaxation mechanisms, and nanoparticulate T1 contrast agents have yet to be approved for clinical use [103,104,105,106]. This flexibility makes iron oxide NPs attractive for detecting specific biological tissues, but their relatively large sizes still impede cell penetration and delivery, while lower values of their magnetic moments require increased clinical uptake to compensate for the poor contrast obtained when compared to gadolinium-based agents. Low efficacy has led to a discontinuation of a number of prominent iron oxide contrast agents in recent years [107,108,109], and currently, only ultrasmall particles (USPIONs) remain in clinical use. Superparamagnetic iron–platinum mNPs have been reported to have significantly better T2 relaxivities than SPIONs and USPIONs [110], while iron mNPs offer an order of magnitude greater susceptibility at room temperature [111,112]. As a result, these are currently agents of significant interest and the topic of much investigation, together with cobalt mNPs, whose very high saturation magnetisation (1422 emu/cm^3^ for cobalt compared to 395 emu/cm^3^ for iron oxide at room temperature) offers a larger effect on proton relaxation, promising improved MRI contrast whilst allowing smaller particle cores to be used without compromising sensitivity [51,55].

#### 1.1.2. Magnetic Hyperthermia

**Figure 2:**
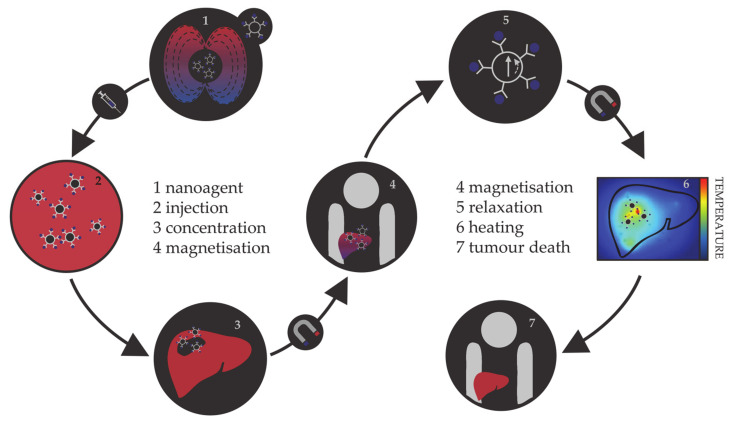
Schematic representation of the principle of magnetic nanoparticle hyperthermia for liver cancer.

Hyperthermia, in terms of medical treatments, is defined as a moderate increase in temperature (to 40–45 °C) sufficient to cause death of tumorous cells whose vulnerability towards heat originates from the poor blood flow and insufficient oxygenation in the affected region [113,114,115]. Initially, hyperthermia treatments used water baths; later, conventional therapies proceeded to noncontact external devices for transfer of thermal energy either by irradiation or by electromagnetic waves (microwave, radiofrequency, ultrasound, or laser sources). However, the realisation of the full clinical potential of hyperthermia treatments was limited due to the inability of heat sources to target tumorous cells efficiently and locally. As the effectiveness reduces steeply with the distance from the source, targeted regions were not receiving enough thermal energy, while maximal temperature gradient was obtained on the body’s surface. However, the biggest shortcoming was the dissipation of energy that was causing serious damage in the healthy tissue situated near the main path of the radiation beam. Altogether, conventional hyperthermia showed no discrimination between targeted tissue and surrounding environment. The growing usage of magnetic nanosystems initiated an efficient solution, where the problem of an external source could now be circumvented by the intravenous administration of magnetic NPs, followed by the use of an alternating magnetic field that results in the localised transformation of electromagnetic energy into heat by means of NP relaxation mechanisms. This targeted approach allows local heating of tumorous cells with minimal impact on the surrounding tissues. The principle of magnetic nanoparticle hyperthermia is presented in Figure 2.

The magnetic hyperthermia nanoagents implemented thus far are mostly magnetite and maghemite NPs [116,117,118,119,120]. However, despite the promising results of preclinical trials, there are many ongoing challenges in making magnetic nanoparticle hyperthermia a universal cancer treatment, including the establishment of optimal limits on the strength and frequency of the applied magnetic field, their correlation with the duration of the treatment, and determination of sufficient NP concentrations [121,122,123,124,125]. As the magnetic gradient decreases with the distance from the source of the applied magnetic field, restrictions on the human-safe magnetic field strengths impose challenges in obtaining the necessary gradients to control the residence time of NPs in the desired area. Additionally, estimates of the magnetic field strengths and gradients based on the hydrodynamic conditions of vascular vessels have shown that the highest effectiveness of magnetic targeting is in the regions of slower blood flow, which are usually near the surface [126,127,128]. Research on the internal magnets implanted in the vicinity of the targeted tissue using minimally invasive surgery is ongoing, and several studies have succeeded in simulating the interaction between an implant and a magnetic agent [129,130,131,132]. In terms of the amount of NPs that can be incorporated in a single living cell, the admissible intake is of the order of a few picograms [133,134,135]. This limit makes it essential that the nanoparticular agents have high magnetic moments, because a relatively small number of particles (between 10^3^ and 10^4^) has to be capable of increasing the intracellular temperature by several degrees, where mNPs have an important advantage over the relatively weak magnetic moments of iron oxides. There are several excellent reviews in the literature on the principles and requirements of magnetic nanoparticle hyperthermia [136,137,138,139,140].

Nanoparticle hyperthermia can also be combined with other therapies to form multimodality treatments and provide a superior therapy outcome [141]. One possibility is to merge it with chemotherapy, where heat can enhance the cytotoxicity of chemotherapeutic drugs or assist the drug uptake by increasing the local blood supply and tissue oxygenation [142,143,144]. Besides the synergy with chemotherapy, the combination of unique magnetic and optical properties in a single NP system leads to multimodal photothermal and thermal photodynamic treatments [145,146,147,148,149]. In nano-photodynamic therapy, mNPs act as photosensitiser carriers that under the irradiation with visible (VIS) and/or near-infrared (NIR) light generate reactive oxygen species (ROS) able to cause tumour degradation. Multiple studies on glioblastoma have shown that the combination of nanoparticulate hyperthermia and photodynamic treatments is quite effective to treat this type of cancer [150]. In nano-photothermal therapy, a nanosized photothermal agent is stimulated by both specific band light and vibrational energy/heat release to selectively target abnormal tissues. A number of magnetic nanomaterials with appropriate optical characteristics have been developed by bringing together a magnetic core and, for example, gold coatings, carbon nanotubes, or graphene, all of which show strong optical absorbance in the NIR optical transparency window of biological tissues. The major advantage of the combined electron–phonon and magnetic relaxations is the significant reduction in the laser power density required for efficient therapies [151,152]. Recently, a pilot study on the initial evaluation of nano-photothermal agents based on gold nanoshells in the treatment of prostate cancer confirmed the clinical safety of this combined therapy [153].

#### 1.1.3. Targeted Drug Delivery

One of the most rapidly developing areas of modern pharmacology is targeted drug delivery, with the aim to reduce the drug intake per dose and prevent exposure of healthy tissue to chemically active analytes. In 1906, Paul Ehrlich introduced the term *magic bullet*, describing the drug capable of locating the causative agent of the disease and providing the adequate treatment without further distribution to unaffected areas [154]. Several decades later, the first drug delivery systems were developed, containing active medical substances attached to the surface of a carrier or encapsulated within the carrier which possesses specific cell affinity contained within molecular vectors and would disintegrate and release the capsulated drug upon contact with diseased cells [155,156,157]. Organic nanosystems (liposomes, micelles, polymer NPs) [158,159,160] together with carbon nanotubes and fullerene NPs [161,162,163] are the most often employed drug carriers, while different hormones, enzymes, peptides, antibodies, and viruses often serve as molecular vectors [164,165,166,167]. Thus far, the available carriers and capsules for targeted drug delivery have each shown several disadvantages, from limited chemical and mechanical stability of organic NPs, over the questionable toxicity of carbon-based systems, to the general susceptibility to microbiological attack, lack of control over the carrier movement and the rate of drug release, and, finally, high cost [168,169,170,171]. Hence, the search for an optimal carrier has shifted direction towards utilising the magnetically induced movement of magnetic nanosystems. Their main advantages are simple visualisation (based on the principles of MRI), easy guidance and retention in the desired area by externally applied magnetic field, and controllable drug detachment triggered by heat released in the variable magnetic field (based on the principles of magnetic hyperthermia). In addition, it is possible to engineer magnetic NPs to either avoid or interact with the immune system in specific ways [172,173].

In common with the previous two applications, most attention has been devoted to iron oxide NPs [174,175,176]. However, limitations of magnetic drug delivery have promoted the search for materials of higher magnetic moments [177] due to the decrease in the magnetic gradients connected to the distance from the source as well as to the fluid hydrodynamics correlating with the depth of the affected tissue—i.e., the same drawbacks as in hyperthermia treatments. So far, a combination of relatively strong magnetic fields with SPION drug carriers has been shown to reach an effective depth of 10–15 cm in the body [178]. Other restrictions relate to the acceptable size of the NPs; first, they have to be below the critical size for optimal magnetic properties, which is a prerequisite to avoid magnetic memory and agglomeration once the magnetic field is removed, and second, they have to be small enough such that after the attachment of drug molecules on their surface they can still effectively pass through narrow barriers [179,180]. The small size implies a reduced magnetic response and hence requires materials of high magnetisation, such as mNPs, rather than metal oxides. Recently, 5–25 nm diameter cobalt–gold mNPs with a core–shell structure and tailorable morphology were synthesised for the purpose of obtaining high-magnetisation drug carriers [181]. The major advantage of the implementation of cobalt within the mNP core is that it has a magnetic moment nearly twice that of iron oxides.

## 2. Features of mNPs

### 2.1. Electronic and Magnetic Properties of mNPs

The proper functionality of mNPs for specific applications depends on their magnetic properties, as well as their biophysical behaviour under physiological conditions. While the latter is most efficiently captured by *in vivo* experiments, insight into the dependence of magnetic properties of nanometre-scale particles on their size, composition, and morphology can be reliably obtained by computer modelling techniques.

Magnetic properties of mNPs can be classified as intrinsic or extrinsic. The former are more important since they are derived from the interactions on an atomic length scale and highly depend on chemical composition and grain size, shape, and crystal microstructure. Additionally, they are much more affected by surface effects and therefore give rise to specific manifestations, such as superparamagnetism, that can only be found at the nanoscale level. These properties include magnetic saturation, anisotropy, and the Curie temperature. 

Intrinsically, classification of mNPs based on the ordering of their magnetic moments corresponds to the classes of bulk metallic materials, and hence there are paramagnetic and ferromagnetic mNPs. Those that are paramagnetic exhibit no collective magnetic interactions and they are not magnetically ordered; however, in the presence of a magnetic field, there is a partial alignment of the atomic magnetic moments in the direction of the field, resulting in a net positive magnetisation. mNPs belonging to the ferromagnetic class exhibit long-range magnetic order below a certain critical temperature, resulting in large net magnetisation even in the absence of the magnetic field. If the diameter of the mNP is larger than the critical value, *D*_C_, coupling interactions cause mutual spin alignment of adjacent atoms over large volume regions called magnetic domains. Domains are separated by domain walls, in which the direction of magnetisation of dipoles rotates smoothly from the direction in one domain towards the direction in the next. Once the diameter falls under the critical value (typically between 3 and 50 nm), mNPs can no longer accommodate a wall and each of them becomes a single domain. Additionally, since each domain is also a separate particle, there can be no interactions or ordering of domains within a sample, and particles do not retain any net magnetisation once the external field has been removed. This phenomenon is known as superparamagnetism. Superparamagnetic mNPs are, as the name suggests, much alike paramagnetic mNPs apart from the fact that this property arises from ferromagnetism. Their normal ferromagnetic movements combined with very short relaxation times enable the spins to randomly flip direction under the influence of temperature or to rapidly follow directional changes in the applied field. The temperature above which the thermal energy will be sufficient to suppress ferromagnetic behaviour is called the blocking temperature, *T*_B_. Below *T*_B_, the magnetisation is relatively stable and shows ferromagnetic behaviour, while for *T* > *T*_B_, the spins are as free as in a paramagnetic system and particles behave superparamagnetically. Blocking temperatures for most mNPs are below 100 K [182,183,184,185], and their behaviour is therefore paramagnetic, as for most temperatures they are only magnetised in the presence of the external field, but their magnetisation values are in the range of ferromagnetic substances. Moreover, the strength of the external field needed to reach the saturation point of superparamagnetic mNPs is comparable to that of ferromagnetic mNPs. 

The highest magnetisation that mNPs can obtain when exposed to a sufficiently large magnetic field is called the saturation magnetisation, *M*_S_. It is the maximum value of the material’s permeability curve, where permeability, *μ*, is the measure of magnetisation that a material obtains in response to an applied magnetic field (total magnetisation of material per volume). It is often correlated with the ratio of magnetisation to the intensity of an applied magnetic field *H*, which is known as the magnetic susceptibility, *χ*, and describes whether a material is attracted into or repelled out of a magnetic field. The magnitude of saturation is a function of temperature; once it is reached, no further increase in magnetisation can occur even by increasing the strength of the applied field. The unique temperature limit at which ferromagnetic mNPs can maintain permanent magnetisation is the Curie temperature, *T*_C_. Notably, when the mNP size is reduced from multidomain to a single domain, the magnitude of *M*_S_ decreases due to the increment in the spin disorder effect at the surface; thus, the *M*_S_ value is also directly proportional to the size of mNPs. 

In almost all cases, magnetic materials contain some type of anisotropy that affects their magnetic behaviour. The most common types of anisotropy are (a) magneto-crystalline anisotropy (MCA), (b) surface anisotropy, (c) shape anisotropy, (d) exchange anisotropy, and (e) induced anisotropy (by stress, for example), where MCA and shape anisotropy are the most important in mNPs. Magneto-crystalline anisotropy is the tendency of the magnetisation to align along a specific spatial direction rather than randomly fluctuate over time. It arises from spin–orbit interactions and energetically favours alignment of the magnetic moments along the so-called easy axis. Factors affecting the MCA are the type of material, temperature, and impurities, whereas it does not depend on the shape and size of the mNP. Morphology effects are included in the shape anisotropy. Stress anisotropy implies that magnetisation might change with stress, for example when the surfaces are modified through ligand adsorption, which means that the surface structure can significantly influence the total anisotropy. Hence, due to the large ratio of surface to bulk atoms, the surface anisotropy of mNPs could be very significant, and the coating of mNPs can therefore have a strong influence on their magnetic anisotropies. Different types of anisotropy are often expressed simply as magnetic anisotropy energy (MAE), which determines the stability of the magnetisation by describing the dependence of the internal energy on the direction of spontaneous magnetisation. It has a strong effect on the values of extrinsic properties.

Extrinsic properties of mNPs are not as essential as the intrinsic. They are derived from long-range interactions and include magnetic coercivity and remnant magnetisation (remanence), which are dependent on microstructural factors, such as the orientation of intermetallic phases.

Magnetic coercivity, *H*_C_, can be described as a resistance of a magnetic material to changes in magnetisation, and it is equivalent to the magnitude of the external magnetic field needed to demagnetise material that has previously been magnetised to its saturation point. Ferromagnetic mNPs that have reached saturation cannot return to zero magnetisation in the same direction once the applied field has been removed, and the magnetic field is therefore applied in the opposite direction. This process leads to the creation of a loop known as hysteresis. Hysteresis loops indicate the correlation between the magnetic field and the induced flux density (*B*/*H* curves). Superparamagnetic mNPs each have only one domain, and no hysteresis loop is obtained when the applied field is reversed. Remnant magnetisation, *M*_r_, is magnetisation left after the magnetic field has been removed. Once the saturation has occurred and a magnetic field is no longer applied, ferromagnetic mNPs will produce an auxiliary magnetic field and resist sudden change to remain magnetised. In contrast, superparamagnetic mNPs will behave as paramagnets with instant need for demagnetisation and negligible *M*_r_. This property allows for ferromagnetic mNPs to gain magnetic memory.

### 2.2. Biomedically Desired Properties of mNPs 

Specifics of the application of interest govern the desired properties of materials used, as was described briefly for the diagnostics and therapy methods in the previous section. In biomedicine, the safety of the treatment towards a patient is the highest priority, and hence superparamagnetic mNPs are preferred because they are magnetised only under the influence of an external magnetic field and quickly demagnetise otherwise, which makes them safer for the human body. This implies that no coercive forces or remanence exist, preventing magnetic interactions between particles and their aggregation, which could lead to adverse problems derived from the formation of clots in the blood circulation system. Saturation magnetisation is also a substantial factor for two reasons: (1) mNPs with high *M*_S_ show a more prominent response to the external magnetic field; (2) high *M*_S_ makes the movements of mNPs more controllable and guarantees efficient response to the magnetic field, implying reduced time of residence and lower required dosages of mNPs. *M*_S_ is dependent on the mNP magnetic moment, size, and distribution, and it is thus important to take them into consideration. An increase in size yields higher *M*_S_, but above the critical diameter, mNPs become ferromagnetic and show undesired behaviour due to the formation of agglomerates and magnetic memory. Moreover, very small diameter sizes are highly desirable to reach regions of limited access; in order to cross the blood–brain barrier, for example, a magnetic core size of *d ≈* 12 nm or less is required. Thus, a suitable balance should be found between the size distribution and magnetic properties. Since mNP-based therapies work by directing the mNPs to a target site using an external magnetic field, magnetic anisotropy is also a very important factor.

Alongside these general requirements that are applicable to all biomedical applications, to enhance the performance of mNPs within MRI diagnostics and hyperthermia therapy it is essential to gain insight into the inherent mechanisms behind their magnetic processes and assess the properties of mNPs and external magnetic field parameters for optimal treatment results.

#### 2.2.1. Relevant Features in MRI

The mechanism of relaxation enhancement directed by MRI contrast agents arises from the dynamic interactions of water molecules with the magnetic centres. Established classical models have significantly contributed to the correlation between the contrast agents’ properties and performance, paving the way to the smarter design of new materials. These models are based on the interpretation of proton–electron interactions between water protons and contrast agents, which is the most important mechanism behind the T1 and T2 contrasts within the contrast-agent-assisted MRI [186,187,188].

For contrast agents consisting of magnetic centre–ligand complexes, interacting water protons are classified into inner-sphere, second-sphere, and outer-sphere protons, each having distinct interaction mechanisms. The inner-sphere mechanism involves direct magnetic centre–water coordination, whereas the second-sphere mechanism describes interactions between the magnetic centre and water protons situated within the second coordination sphere through hydrogen bonds. The outer sphere includes the influence of the magnetic centre on the translational diffusion and rotational motion of the remaining bulk water protons [97,189]. However, for contrast agents consisting of magnetic particles, the direct coordination of water is atypical, and the largest share of the nuclear magnetic relaxation of water protons in solutions (or suspensions) of magnetic particles arises from pure magnetic interactions at the molecular level. In essence, this amounts to the reformulation of the outer-sphere mechanisms from a single metal atom complex centre to the solution of mNPs.

Superparamagnetic mNPs with large magnetic susceptibility produce local magnetic fields under the influence of an external magnetic field. As a result, the local magnetic field causes a perturbation in the motion and relaxation of nearby water protons. Hence, the relaxation is induced by the fluctuating dipolar interaction between the nuclear magnetic moment of the water proton and the global magnetic moment of the mNP, just as in the case of classical outer-sphere theory. This reformed outer-sphere theory now describes the change in the relaxation of solvent protons as the water molecules diffuse into the neighbourhood of a solute particle and start interacting with its magnetic dipolar moment.

This type of local perturbation shortens the T2 relaxation time, also known as spin–spin relaxation, of water protons during water diffusion. The mNP contrast agent catalytically relaxes water protons at the particle–solvent interface emanating in T2-weighted MRI images. The extent to which the mNP contrast agent affects the relaxation rate of tissue water can be quantitatively characterised by its transversal relaxivity, $r_{\mathrm{CA}}$, and the final relaxation rate of the tissue, $r_{2}$, is given by:

(1) $$r_{2}=\frac{1}{T_{2}}=\frac{1}{T_{2}^{o}}+r_{\mathrm{CA}}\left[\mathrm{CA}\right].$$

mNP relaxivity is thus defined as a proportionality constant between the induced increase in the relaxation rate of the tissue and the concentration of the contrast agent, [CA]. By definition, low doses of high-relaxivity contrast agents provide an equivalent contrast magnification as higher concentrations of contrast agents with inferior $r_{\mathrm{CA}}$. Every tissue has an inherent relaxation rate, $1/T_{2}^{o}$, and in order to generate an observable contrast, the relaxivity of the contrast agent should be at least at 10% of the inherent rate. 

Outer-sphere theory for T2 relaxivity has developed an expression for the dependence of relaxivity performance of superparamagnetic mNPs on their intrinsic properties—saturation magnetisation and the effective radius:

(2) $$r_{2}=\frac{1}{T_{2}}=\kappa_{\gamma }V_{\mathrm{eff}}M_{s}^{2}\frac{r^{2}}{D\left(1+l/r\right)},$$

where $\kappa_{\gamma }$ is the constant derived from the gyromagnetic ratio of solvent protons, $V_{\mathrm{eff}}$ is the effective volume fraction ($V/\;\left[\mathrm{CA}\right]$), $M_{s}$ is the saturation magnetisation, r is the radius of the magnetic core, l is the thickness of impermeable mNP coating, and D is the diffusion coefficient of water molecules. Viewed simplistically, an increased $M_{s}$ value is reflected in the increased response of mNP contrast agents to be magnetised by the external field, resulting in the higher $r_{2}$ relaxivity. Similarly, improved relaxivities can be achieved with a larger magnetic core radius. As the proton relaxation occurs mainly at the interface of the mNP and surrounding aqueous environment, nanoparticle coatings also influence the rate of T2 relaxation through the coating thickness.

T2 relaxation processes occur through three mechanisms. The first mechanism, known as Curie spin relaxation, arises from the dipolar interactions between water protons and a large magnetic moment of unpaired mNP electrons. It depends on the strength of the applied magnetic field and is a function of the size and water diffusion time, $r^{2}/4D$. This mechanism is prominent for small-sized mNP contrast agents under strong fields, while it decreases rapidly for larger mNPs where T2 relaxation is mostly dominated by the remaining two mechanisms: dipole–dipole coupling between metal ions and hydrogen nuclei and scalar or contact relaxation processes. Hence, a primary factor affecting $r_{2}$ is the mNP-generated inhomogeneity, which depends largely on the magnetisation of the contrast agent. In general, more efficient inhomogeneity originates from materials with higher saturation magnetisation; such materials can also influence a greater volume of surrounding water. However, the effective magnetisation value of mNPs is often several times lower than that of the bulk counterpart, caused primarily by an increased magnetic anisotropy. Due to the presence of magnetically ‘dead’ or tilted layers of atoms on the mNP surface, the surface atomic spins are largely canted, thereby enhancing the MAE and reducing overall magnetisation. This effect is especially pronounced in small particles, owing to their high surface-to-volume ratio. Additionally, MAE is also affected by the mNP morphology and surface interactions. For particles of the same volume, a reduced shape and surface anisotropy lead to the spin state similarity between the surface and core, thereby increasing the magnetisation. Contact interfaces between two different magnetic phases, for example in the form of core–shell NPs, often provide an additional source of anisotropy (exchange anisotropy), resulting in a slight reduction but also in stabilisation of magnetisation and improved coercivity. Moreover, changes in the magnetic moments of surface atoms can be enforced through surface functionalisation. 

Diffusion dynamics of water molecules in the magnetic field gradients is another important factor for effective $r_{2}$ rates. It is characterised by the number of water molecules that have diffused into close proximity of the interface with the contrast agent and their residency time within that region. mNPs with large magnetic moments have a stronger tendency to form dipole interactions with water protons, to form a larger area of influence, and to provide a greater possibility of relaxing the diffused water molecules. Certain coatings can also be beneficial in this matter, while others may hinder water diffusion or prolong water residency within ligand pockets, reducing the image contrast. Coatings forming a hydrophilic mNP surface favour diffusion and retention of water molecules in the mNP outer-sphere. Finally, fine-tuning of the thickness, charge (ligands rich in π-electrons create small magnetic fields increasing inhomogeneity), and porosity of the coating allows for optimised water accessibility and residency. 

Hence, the dynamic interactions behind the relaxation mechanisms depend predominantly on the magnetic properties of mNP contrast agents, which, in turn, are attributed to a large extent to the mNP structural features. Relaxivity of superparamagnetic mNPs can be enhanced not only via their magnetic properties, but also through the coating optimisation. The last parameter directly included in the equation, without specifically considering its effect on the magnetic properties, is the diameter of the magnetic core. From the relationship, the $r_{2}$ value can be increased by increasing the core size. However, biomedical applications are limited by the superparamagnetic size limit. Within this critical diameter, theoretical studies have identified three mNP size regimes depending on the $r_{2}$ rate trends: the motional average regime (MAR), the static dephasing regime (SDR), and the echo-limiting regime (ELR) [190]. With the growing mNP size in the MAR regime, relaxivity increases, reaching a plateau in the SDR regime. With any further size increment, mNPs fall in the ELR regime where $r_{2}$ steeply decreases. Accordingly, the highest $r_{2}$ is achieved for mNPs within the SDR, but nanoparticulate contrast agents employed thus far have been within the MAR regime to limit the NP aggregation through magnetic interactions [191,192].

Finally, in nanoparticle-based imaging, higher mNP concentrations lead to an improved signal-to-noise contrast, at a cost of a toxicity trade-off. Therefore, even though it does not directly affect the relaxivity constant, mNP concentration is one of the most important parameters in the development of nanoparticulate contrast agents, with the objective of obtaining sufficient contrast at the safest (lowest) mNP concentrations. Studies on SPIONs have consistently shown significant enhancement in the MRI signal when the concentration of Fe progresses from 9 to 100 µg/mL [193,194,195], and the minimum concentration found to produce marked hypointense signals was evaluated at or above 22.4 µg/mL [196]. Moreover, upon 24 h incubation, Mn-doped SPIONs displayed no appreciable cytotoxicity even at the highest metal ion concentrations, indicating good biocompatibility [193]. Furthermore, the sensitivity of the imaging technique must also be taken into account. So far, the most sensitive detection experiments on mNP imaging have demonstrated sensitivity to particle concentrations in the picomolar and tens of picomolars range. For example, the tissue T2 contrast is clearly distinguished at gold-coated Co mNP concentrations as low as 50 pM [55]. This detection threshold is 7 orders of magnitude better than those for monocrystalline iron oxide particles, whereas the T2 relaxivity per-particle concentration is improved up to 5 orders of magnitude compared to both magnetite and Fe/Au NPs [197,198], which translates into lower mNP concentrations for efficient MRI contrast.

#### 2.2.2. Relevant Features in Hyperthermia

The heating efficiency of the magnetic nanoparticle hyperthermia process depends on the power dissipated by the mNP following the application of an alternating magnetic field, and it is often quantified through the specific absorption rate (SAR). SAR is the rate at which the power is absorbed by a volume of dielectric material, such as biological tissue, exposed to electromagnetic radiation (or another source of energy). It is often mentioned interchangeably with the specific heating power (SHP), which is defined as the thermal power per unit mass dissipated by the magnetic material and accurately describes a material’s heating efficiency. The interconversion of magnetic field energy into heat by mNPs can arise through three mechanisms: eddy currents, hysteresis, and relaxation processes. Eddy currents are loops of localised electric current induced by the varying magnetic field. Their existence depends on how resistant the material is towards current heating, and they are usually present in bulk crystals. The prevailing heating mechanism in multidomain mNPs is hysteresis loss, while single-domain superparamagnetic mNPs have high electrical resistivity, and such power loss is thus negligible compared to that originating from relaxation processes. Relaxation mechanisms have two modes: following the removal of the external magnetic field, magnetic moments relax either through the motion of the internal spin (Néel relaxation) or by the rotation of the mNP around its own axis (Brownian relaxation). Either relaxation of the magnetic moment back to the initial position releases thermal energy and induces local heating due to friction. If the mNP undergoes Néel relaxation, heat is dissipated by the rearrangement of atomic dipole moments where internal friction causes phase lagging between the applied field and magnetic moments. If the mNP relaxes through a Brownian relaxation mechanism, the power loss arises from the shear stress in the surrounding medium. Additional power loss can occur due to the physical relaxation of the liquid. 

Quantification of the specific heating power of mNPs is derived as:

(3) $$\mathrm{SHP}=\frac{1}{2}\omega \mu_{0}\chi_{0}H^{2}\frac{\omega \tau }{1+\omega^{2}\tau^{2}}$$

where ω is the angular frequency of the external magnetic field and equals ω=2πf, $\mu_{0}$ is the magnetic permeability constant of free space, $\chi_{0}$ is the magnetic susceptibility, and H is the magnitude of the external magnetic field. The fractional term leads to the maximal power at ωτ=1; in this case, the matching ω is called a critical frequency. An estimated goal for the material development in this field is an SHP of the order of 1000 Wg^−^^1^, based on the amount of mNPs that can be incorporated by a single living cell [16]. The critical factor is hence the relaxation time of mNPs, τ, resulting from prevailing heating mechanisms.

Relaxation time can be defined as the time needed for magnetic moments of mNPs to vanish once the external magnetic field has been shut off. For Néel relaxation, the time constant of the external magnetic field is short enough so that magnetic moment alternates from parallel to antiparallel orientation and back without a change in the physical orientation of the particle. As excitation occurs against the anisotropy energy barrier, the process strongly depends on mNP volume and anisotropic properties, and it is not influenced by the conditions of the surrounding environment. The relaxation time for Néel relaxation,$\;\tau_{N}$, can be described by:

(4) $$\tau_{N}=\frac{\tau_{0}}{2}\sqrt{\pi \frac{kT}{KV}}e^{KV/kT}$$

where $\tau_{0}$ is the characteristic relaxation time and equals $\tau_{0}=1/f_{0}$ ($f_{0}=10^{9}-10^{13}$ s^−^^1^), k is the Boltzmann constant, T is the temperature, K is the anisotropy constant (includes all sources of anisotropy), and V is the volume of the mNP. KV is somehow equivalent to an activation energy that has to be exceeded by the thermal energy, kT, to overcome the inherent magnetic anisotropy energy barrier. In contrast, Brownian relaxation prevails when magnetic anisotropy is sufficient to overcome inertial resistance, in which case the external magnetic field causes rotation of a whole particle with the magnetic moment remaining fixed relative to the crystal axis. The Brownian relaxation time is highly dependent on the hydrodynamic properties of both mNP and surrounding medium, such as viscosity of the fluid and hydrodynamic volume of mNP, which includes any surfactant layer added for colloidal stability. The equation that describes Brownian relaxation time, $\tau_{B}$, is:

(5) $$\tau_{B}=\frac{3\eta V_{H}}{kt}$$

where η is the viscosity of the medium and $V_{H}$ is the hydrodynamic volume of mNPs. When these two processes are simultaneously involved, the effective relaxation time, which describes the energy transfer rate, takes both into consideration, and the final expression for the relaxation time is:

(6) $$\frac{1}{\tau }=\frac{1}{\tau_{N}}+\frac{1}{\tau_{B}}.$$

Overall, the shorter of the two dominates the effective relaxation time—Néel for small particles in viscous solutions and Brownian for particles with large hydrodynamic volume in an environment of lower viscosity. The exact division between mechanisms depends on the anisotropy constant of each material. In magnetic nanoparticle hyperthermia, mNPs are embedded in a tumorous tissue and internalised by cancer cells, either by adhesion to the cell walls or in the form of the restraint of movement provided by cell plasma. Because of this immobilisation, Brownian mechanism is damped to the maximum and Néel relaxation occurs almost exclusively [199].

mNPs can be remagnetised only after their relaxation is completed; therefore, the frequency of the external field has to match the relaxation time in order to obtain efficient heating rates. SHP is maximised under the condition ωτ=1, and since typical Brownian relaxation times in systems where this mechanism dominates over Néel relaxation are around 10^−^^5^ s, effective frequencies would need to be 10^5^ rad s^−^^1^ (15 kHz), which is much lower than any frequency reported in hyperthermia studies (100–300 kHz range) and therefore favours relaxation times of 10^−^^6^ s or below [139,200]. This confirms Néel relaxation as the predominant heating mechanism in magnetic nanoparticle hyperthermia and accentuates the need for magnetic anisotropy energies that significantly exceed the thermal energy (KV≫kT); if the anisotropy constant is not satisfactory, no noteworthy heating is expected. Therefore, SHP depends on the size, magnetisation, and anisotropy of mNPs, as well as on the characteristics of the applied external magnetic field.

From the equation for $\tau_{N}$, MAE is an important factor in enhancing the Néel relaxation time. However, within the limitation of ωτ=1 to maximise SHP, an enhancement of the relaxation time may not always yield higher SHP values, and the frequency of the external magnetic field must be chosen accordingly [139]. Only through that correlation can efforts to increase the MAE of mNPs yield a higher heating efficiency and, ultimately, by satisfying the ωτ=1 condition, allow for the use of lower frequencies of the applied magnetic field [201].

Magnetic nanoparticle hyperthermia involves the excitation of mNPs suspended in a fluid medium using the external magnetic field, meaning that parameters of the magnetic field itself should also be optimised to obtain the desired results. Changes in frequencies and amplitudes directly and proportionally influence the heating power of mNPs, where SHP increases rapidly with the increase in the strength of the magnetic field. Enough heating power must be generated for the destruction of cancerous tissue, while at the same time the frequency and strength of the applied magnetic field have to be safe for the human body. These requirements force strict limitations on the frequencies to range from 0.05 to 1.2 MHz and the magnetic field strengths to range from 0 to 15 kA m^−^^1^. Higher values would lead to serious problems, such as aggregation of mNPs causing embolisms, while lower frequencies would stimulate skeletal, cardiac, and peripheral muscles and trigger arrhythmias. Amplitudes are usually in the range of 5–30 kA m^−^^1^ and external magnetic fields with $H_{0}\times f$ less than 4.85 × 10^8^ A m^−^^1^ s^−^^1^ have been approved for successful use in humans [202].

Theoretically, SHP cannot be controlled by the mNP concentration because they show no obvious interdependence; however, higher mNP concentrations provide more efficient hyperthermia performance as less time is required to reach optimal temperatures. The condition of sufficient heat generation by mNPs to sustain tissue temperature of at least 42 °C for ~30 min was achieved in different studies by using SPION concentrations in the 0.1 to 400 mg/mL range, significantly higher than when used as T2 contrast agents [116,203,204,205,206]. Nonetheless, according to several recent reports, it was surprisingly observed that SHP and SAR values can be affected by mNP concentration, contrary to the invariance for 6–300 mg/mL measured for mNPs dispersed in water [207]. The physical principle of SHP oscillations was explained by the interparticle magnetic dipole and mutual potential energy interactions, dividing the dependence into four concentration regions. In the region of the lowest concentrations (<0.1 mg(Fe)/mL), the nanofluids showed the highest SHP; in region 0.1–1.0 mg(Fe)/mL, a remarkable drop in SHP was observed; at 1.0–10 mg(Fe)/mL, the SHP was increased again; and at the highest concentrations (10 mg(Fe)/mL or more), the SHP was re-decreased [135,208].

Different combinations of superparamagnetic materials and biocompatible ligands have been investigated, but achieved efficiencies are far from ideal and further efforts are directed to find the best nanocomposites. The most important advance in the last 10 years was the commencement of the first-ever clinical studies of therapeutic hyperthermia induced by magnetic NP heating [121]. The study successfully demonstrated that magnetite NPs can be safely applied in the treatment of brain tumours and that hyperthermic temperatures are achieved. Magnetite and maghemite NPs exhibit (up to this point) medium heating efficiency with little or no control of temperature changes when compared to other magnetic materials, but their biocompatibility, nontoxicity, and ability to escape the reticuloendothelial system makes them preferred candidate NPs for magnetic hyperthermia. However, the relatively low heating rates of conventional SPION hyperthermia agents [137] (less than 100 W g^−^^1^ for 400 kHz frequency and 10 kA m^−^^1^ magnetic field strength) unfortunately often require a high mNP concentration, which would not only result in potential toxicity but also complicate the monitoring of the progress of tumor response using imaging tools, thus promoting the implementation of mNPs with higher specific magnetisation. Many efforts have been made in order to improve the properties of magnetite NPs; a valuable strategy was to increase their MAE by the total or partial substitution of Fe^2+^ ions by Co^2+^ ions. Cobalt ferrites have shown comparatively high thermal and oxidative stability with higher suitable MAE and consistently large heating effects with SHP values reaching 720 W g^−^^1^, which is significantly higher than the rates reported for the iron oxide NPs (22–200 W g^−^^1^ [209,210,211]). The current focus of research is on cobalt mNPs to further improve the heating efficiency in hyperthermia therapy.

#### 2.2.3. Relevant Features in Drug Delivery

Magnetic targeting of drug delivery carriers is based on the attraction of mNPs induced by an external field. For the mNP drug carrier to be efficiently trapped in the magnetic field at the targeted site, the gradient of the magnetic field has to exert a sufficient translational force on the particle–drug complex. This magnetic force, $F_{mag}$, can be expressed as:

(7) $$F_{mag}=\left(\chi_{2}-\chi_{1}\right)V\frac{1}{\mu_{0}}B\left(\nabla B\right)$$

where $\chi_{1,2}$ are the magnetic susceptibilities of the medium and the mNP, V is the volume of mNPs, $\mu_{0}$ is the magnetic constant, *B* is the strength of the magnetic field, and ∇B is the field gradient. The susceptibility of the biological medium is usually very small compared to that of mNPs and can be disregarded. From the expression, the effective capture of the mNPs depends on the magnetic properties and volume of the particles, as well as on the parameters of the applied magnetic field. As the magnetisation of the mNP decreases, the ability of the magnetic field to capture and direct them also decreases. Correspondingly, insufficient field strengths and gradients have limited penetration depth and generate weak translational force. Estimations from experimental studies and extended theoretical investigation of the hydrodynamic conditions of mNP drug carrier targeting have indicated that a minimal field strength of 200–700 mT is required at the target site with gradients along the *z*-axis of approximately 8–100 T/m depending on the flow rate [126,127].

## 3. Computational Modelling of mNPs

For the optimal performance of mNPs in biomedical applications, it is important that they are biocompatible and nontoxic and have colloidal stability, opportune surface modification, an appropriate particle size, and, above all, adequate magnetic properties. The dependence of magnetic properties on the structural parameters of mNPs, namely their size and shape, is well known, with paramount examples in the literature [212,213,214,215,216,217,218]. However, the determination of this correlation is by no means easy or exempt from complications. Even with advances in the control over the synthesis and characterisation, an ever-present distribution of mNP sizes ultimately prevents the assignment of specific magnetic features to a particular size or morphology. Furthermore, capping agents increase the complexity of the system, adding even more uncertainty to the assignment of the origin of specific magnetic behaviour. These impediments in the determination of a specific connection between the mNP morphology, coating, and magnetisation are a substantial limitation in the development of property-tuning strategies, which are an ultimate tool in the engineering of mNPs for specific applications. It is in this sense that computational modelling becomes a powerful complementary technique to experiment, by unravelling the connection between the properties of mNPs and their structural factors.

The modelling of isolated monometallic mNPs is only limited by the availability of sufficient computational resources, and quantum mechanical calculations have become the standard technique to obtain properties of clusters and smaller mNPs of up to a few hundred atoms [219,220,221,222,223,224,225,226,227]. The acquisition of resources for quantum simulations can be problematic for larger mNPs with more realistic diameters that are closer to application-relevant sizes. For simulations of such mNPs with hundreds or thousands of atoms, approximate theoretical models have been designed, based on classical interatomic potentials with parameters that were fitted either to the experimentally captured properties or to the descriptors obtained through quantum density functional theory (DFT) calculations [228,229,230,231,232,233]. It is the quality of these parameters, as assessed by their ability to estimate closely the values of targeted data, that ultimately determines the accuracy of the results obtained from molecular simulations. Despite the success of classical molecular dynamics (MD) in describing the behaviour of large mNPs, the need to predefine fixed interaction potentials in the form of a force field remains a significant challenge for metallic species and their interaction with different capping agents [234,235,236,237]. The lack of the description of bond breaking/forming at the MD level is also hampering any investigation of the mNP reactivity. Additionally, even after the sufficient potentials for a specific system of interest have been elaborated, changing a single species provokes enormous efforts to suitably reparameterise the potential energy function. As a result, systematic studies are a tour de force if consistent potentials are not already available. However, the advent of highly parallelised supercomputers has gone side-by-side with the progress in *ab initio* DFT methods, expanding the initial studies of small gas-phase metal clusters to investigations of large mNPs and their interaction with protective coatings [238,239,240,241,242,243].

This review focuses on the computational modelling of cobalt mNPs as tackled mostly by means of DFT calculations, which has aided insight into the link between their structural and magnetic properties. It is our aim to show that these studies can provide important information to allow reliable predictions of mNP performance within biomedical applications. They provide a useful gateway before attempting a rational, engineered tuning of the magnetisation of Co mNPs, and they may result in a sound modelling approach for magnetic mNPs of varying compositions as they rely only on *ab initio* inputs of physical constants. Coating and alloying effects are also contemplated, as changes in the magnetic behaviour can be induced by an active interplay between the surfactants and mNPs or between different metallic phases.

A physical description and expressions of observed NP phenomena (magnetic relaxation, heat dissipation, etc.) helped to identify factors affecting material capabilities and the treatment outcomes. Incorporation of *ab initio* models in the research efforts to optimise these factors within magnetic nanosystems could hence facilitate tuning strategies of application-specific properties to acquire the maximum treatment efficacy, and DFT results are often implemented in extended Monte Carlo simulations or analytical and numerical models to quantify magnetic field response, proton relaxation, or heat transfer [244,245,246,247]. However, it is important to note that the optimum property settings to generate maximum relaxation or heating through analytically developed expressions cannot ensure clinical suitability, as the optimisation process is often based on a number of assumptions that may deviate in real-life applications [248]. Even *in vivo* and *in vitro* studies sometimes give conflicting results [249,250,251], and DFT predictions and accompanying optimisation models must hence be verified by clinical data.

### 3.1. Monometallic mNPs

#### 3.1.1. Cluster Model

The simulation and description of nanosized metallic systems essentially begin by modelling clusters with a small number of atoms. Quite often, exhaustive efforts to define the cluster structure belonging to the global energy minimum of a certain atomic size (note that the number of possible configurations grows exponentially with the number of atoms) can be simplified by implementing a molecular dynamics simulation. Rives et al. [252] and Rodríguez-López et al. [253] have captured corresponding growth structures for small Co clusters using different potential parameters. Both found an icosahedral growth pattern for the global minimum energy structure with hcp and fcc structures dominating in particular sizes, whereas for the second isomer, distorted icosahedral structures were generally obtained. However, MD-based methods lack the electron-level description necessary for the determination of magnetic behaviour and bond forming and breaking, and hence their use beyond the identification of suitably stable structures is rather limited. Hence, knowledge of electronic structures obtainable through quantum mechanical methods, such as those based on the DFT, is required.

**Figure 3:**
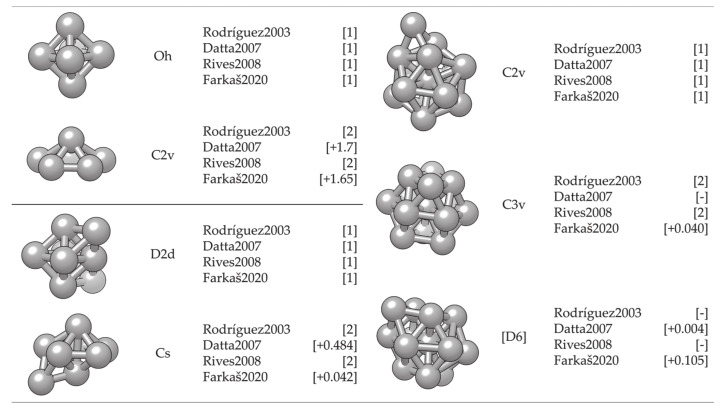
Agreement between most stable cluster structures of 6-, 8-, and 14-atom Co as predicted by DFT [239,254] and MD [252,253] simulations. For DFT studies, energy difference between the structural isomers is given in the square brackets in eV.

**Figure 4:**
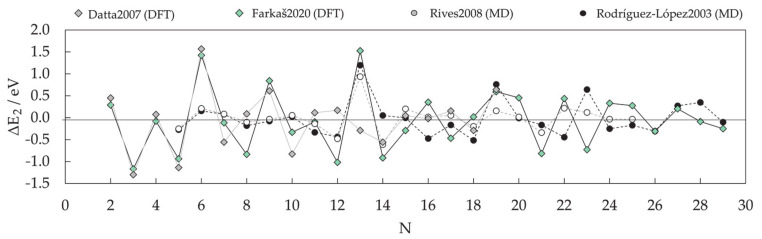
Relative energy stability of successive Co cluster sizes expressed as a second difference in total energy, ΔE_2_, as predicted by DFT (Datta (2007) and Farkaš (2020) [239,254]) and MD (Rives (2008) and Rodriguez (2003) [252,253]) simulations.

DFT results for a number of chosen morphologies will in the majority of cases show good agreement with the MD trends, and the magnetic properties or reactivity behaviour of those structures can be investigated further on the quantum level. The MD-DFT agreement in the stability trends of a convenient number of shapes (convenient in respect of DFT computational cost and exploration of the global minimum well) for each cluster size in the case of Co clusters can be seen in multiple studies, with a few examples shown in Figure 3 [239,253,254]. The second energy difference in the total energy of successive cluster sizes also captures the ability of DFT calculations to reproduce the stable morphologies across the range of cluster sizes (Figure 4).

Small clusters efficiently capture the large surface-to-volume ratio effects on the magnetic and electronic properties, but these observables generally show strong oscillations with the number of atoms per cluster. Such small clusters are known as systems where every atom counts, and an estimation of the properties of a particular cluster size from the characteristics of the neighbouring sized clusters is often misleading. Fortunately, electronic structure features for the couple-atom systems are easily obtainable within the DFT approaches [255,256,257,258,259,260,261].

Because of the property oscillations, which are also heavily dependent on the type of metal atoms that constitute the cluster, a universal description of the progression of magnetic moment, or any other property, does not exist. The closest researchers have come to a unifying picture of metal clusters is the closed-shell model, which is manifested through the filling of electronic shells resulting in clusters of extraordinary stability [262,263,264]. The numbers of electrons corresponding to closed electron shells in metal clusters are 8, 20, 40, 58, etc. This model can often be extended to clusters with geometrically closed shells that often adopt ‘perfect’ structural shapes, such as 13-atom icosahedron. However, the closed-shell model cannot account for a large number of cluster sizes and their structural isomers that are between the clusters with a relevant number of atoms sufficient to form closed electronic or geometric shells. This rich variety of conformational isomers, which often exists within a narrow energy range, and the unique set of properties for each added atom translate into a rather complex issue in the electronic and magnetic structures for small metal clusters [265].

**Figure 5:**
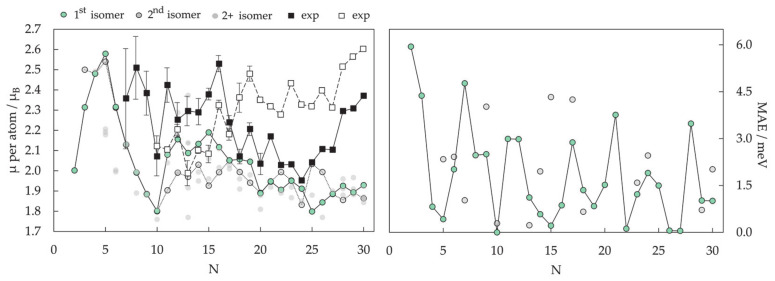
Experimental data are taken from Knickelbein (2006) [270] (■) and Xu (2005) [271] (□), DFT data from Farkaš (2020) [239]. Legend is the same for both graphs.

Magnetic properties, namely magnetic moments per Co atom and magnetic anisotropy energies of small Co clusters (2 ≤ N ≤ 30), are shown in Figure 5. Anything better than a general agreement between experiment and theory is hardly achievable, primarily owing to the deficiency in DFT treatment of the orbital moments, which can be rather large in clusters, in contrast to the bulk metal where they are strongly suppressed [266]. Recent findings of orbital moments in small Co clusters of an order of magnitude higher than those in the bulk further confirm this [267]. Additionally, experimental measurements of magnetic moments also suffer from rapid decrease in the cluster beam intensities, thermalisation, and changes in the direction of magnetisation in response to thermal fluctuations [268,269,270,271]. Inconsistencies in the experimental and computed trends can be improved for intermediate sizes (10 ≤ N ≤ 25) by introducing magnetic moments of the second most stable isomer, confirming the possible coexistence of different isomers in the cluster beam, which was proposed experimentally [268].

Generally, clusters in this size range show significantly increased values of the magnetic moment and MAE compared to the bulk counterparts (1.72/1.64 μ_B_ and 27.2/1.7 μeV MAE per atom for hcp/fcc Co bulk), but the degree of increase is size-dependent. Nevertheless, the differences between magnetic properties of Co clusters and bulk are more a matter of degree than a matter of type, as they are both composed of atoms whose electronic character (i.e., number of unpaired electrons) is responsible for their magnetism. Only in rare instances do metal clusters and metal bulk phases show inverse behaviour [272]. The degree of the cluster’s magnetic character depends on the spin coupling of composing atoms, and, as seen in Figure 5, no obvious magnetic moment or MAE trends exist as the cluster size progresses towards a couple of tens of atoms.

**Figure 6:**
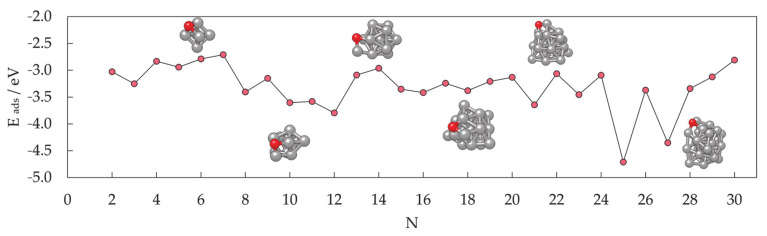
DFT-calculated adsorption energies for single-atom oxygen adsorption on small (2 ≤ N ≤ 30) Co clusters.

In contrast, the chemical properties of small clusters are a combination of the reactivity of bulk and molecular matter, and any general conclusion cannot be simply extrapolated from the bulk behaviour. Similar to the magnetic properties, small and medium clusters show different reactivities that do not vary smoothly with size. Oscillations in the adsorption energies of oxygen on the small Co clusters in the 2 ≤ N ≤ 30 range are shown in Figure 6. At instances, groups of cluster sizes with resembling geometries, such as N = 4–7 or N = 15–19, also show similar adsorption behaviour for atomic adsorbates.

However, two different geometric forms of the cluster of a single size often have different reactivities, similarly to the distinct properties of molecules with the same elemental composition but different conformation (chemical isomers). For example, the computed adsorption energy of an oxygen atom on the six-atom cluster with O_h_ symmetry is −2.79 eV, whereas the same quantity on the six-atom cluster with C_2v_ symmetry is only −1.29 eV. Similarly, 18-atom clusters with icosahedral and hexagonal geometries yielded *E*_ads_ of −2.88 and −3.38 eV, respectively. It is also not unusual for several cluster sizes and morphologies to induce dissociation of certain molecules, whereas the rest of the cluster systems facilitate plain adsorption [273].

These observations have led to the formulation of what is today known as the nonscalable regime, where chemical and physical properties of clusters cannot be predicted through size-correlated trends but are instead completely independent for each number of atoms. Any system showing such autonomous behaviour and belonging to the series of sizes where trends, if any, are captured with great difficulty, should never be referred to as a metal NP, but rather as a metal cluster or a nanocluster, and as such cannot represent realistic behaviour of nanoparticles.

#### 3.1.2. Surface Model

Metal clusters have, nevertheless, been employed as models for metal surfaces and NPs owing to the confined number of atoms, which was easy to simulate by employing the most sophisticated and accurate quantum methods [274,275,276]. However, even though these models were useful for insights into the adsorbate–metal atom interactions, a cluster representation results in an increased number of undercoordinated metal sites, which often translates into unrealistic surface or NP electronic properties and chemical activities [277,278,279]. Consequently, cluster representations have been abandoned, giving place to the extended slab models obtained at the extent of the metal surface periodicity.

The periodic slab model has quickly become the standard for theoretical studies of surface chemistry, and it was believed that a complete description of mNPs can be gained through such simulations if mNPs are considered as a sum of discrete facets [280,281,282,283,284,285]. For example, for those Co cluster sizes with hexagonal symmetry which showed sufficient surface expansion so that the atomic adsorbate can bind to the cluster solely through the facet sites without interacting with any of the undercoordinated atoms (22 ≤ N ≤ 30), the calculated facet adsorption strength (between −3.06 and −3.54 eV) coincides with the adsorption on the extended slab system (*E*_ads_ = −3.12 eV). Since the chemical reactivity of facets of relatively small metallic clusters can be envisioned by employing extended slab models, this should also hold for large mNPs with significantly larger surface areas. Nevertheless, there is still a high level of uncertainty when considering these models as reliable representations of the changes in the electronic properties of mNPs upon chemical adsorption.

**Figure 7:**
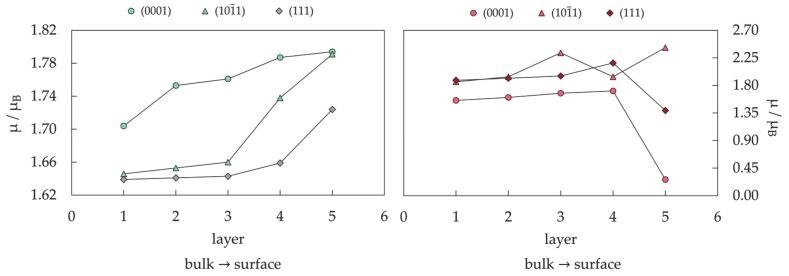
Magnetic moment per Co atom, µ, as a function of the atomic layer within the extended slab models of hcp $\left(0001\right)$, hcp $\left(10\bar{1}1\right)$, and fcc $\left(111\right)$ surfaces of metallic cobalt—**left**: bare surfaces, **right**: oxidised surfaces. Layers are numbered going from bulk to the surface, 1 being the bulk-like inside layer and 5 being the top-most surface layer.

For cobalt, both fcc- and hcp-bulk phases can be a basis for the mNP construction. Hcp-built mNPs are known to be tiled by the $\left(0001\right)$ and $\left(10\bar{1}1\right)$ as the most prominent surfaces, while the $\left(111\right)$ surface dominates the mNPs of the fcc framework. Average magnetic moments of slab surface models are naturally going to show values closer to the bulk magnetisation than to the moments of cluster systems considering the complete absence of any undercoordinated sites. However, layer-by-layer progression of magnetic moments from the top-most surface atoms towards the bulk-like inner atoms of the slab model is often taken as an indication of the shell-to-core progression of magnetic behaviour of larger, facet-saturated mNPs. Magnetic moments of different layers are shown for the three populated surfaces in Figure 7 (left). A 5–8% reduction in the magnetic moment of Co atoms is captured when going from the surface to the inner, bulk-like atomic layers. An obvious deficiency in slab representation of shell-to-core progression of magnetic moments is the planar contact point between the atoms placed in different atomic layers as opposed to the radial distribution of atoms within the real mNP.

Upon adsorption of a full monolayer (ML) of oxygen (where full monolayer or 1.00 ML is established when the number of interacting atomic adsorbates corresponds to the number of Co atoms in the surface layer), the distribution of magnetic moments between the layers significantly changes (Figure 7, right). Hcp $\left(0001\right)$ and fcc $\left(111\right)$ surfaces are flat in the sense that the closest Co atoms are levelled within a straight plane and adsorption takes place monotonously on top of the surface, whereas the hcp $\left(10\bar{1}1\right)$ surface has a row-like arrangement and allows channel-driven adsorption or even stimulates the subsurface adsorption with minimum energy cost. This significantly influences the induced change in the magnetic moments of surface atoms—for the $\left(0001\right)$ and $\left(111\right)$ surface, the top-most surface Co atoms experience a reduction in magnetic moments. In the case of the $\left(0001\right)$ surface, an 85% decrease in magnetic moment is observed, with a 5–10% reduction in the remaining atomic layers. On the $\left(111\right)$ surface with lower packing density, there is a 20% decrease for the first-layer atoms and a 15–30% increase in the inner slab layers. Magnetic moments of the top-most $\left(10\bar{1}1\right)$ surface atoms, on the other hand, have 15–40% higher values after adsorption, and a similar rate of increase is observed for the third atomic layer upon the in-channel incorporation of adsorbate atoms.

However, the effect of adsorption on the magnetic moments is a complex, site-dependent issue, and different atoms within the same layer might experience varying cumulative effects of adsorbate atoms or molecules [286,287,288]. Atom-decomposed properties can hence reveal more details on adsorbate–surface coupling and the magnetic nature of the oxidative layer. For oxidative adsorbates, such as atomic oxygen, saturation of the metallic surface can easily lead towards the formation of a metal oxide skin [289,290,291,292,293,294]. Metal oxides often show significantly distinct magnetic properties from their parent metal materials, which can be unfavourable for many applications.

**Figure 8:**
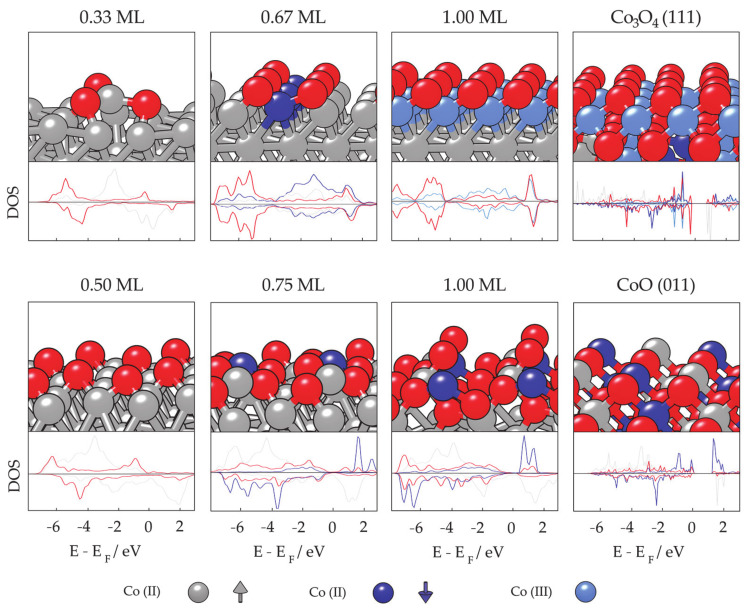
Optimised magnetic orderings for different oxygen coverages on hcp Co $\left(0001\right)$ (**top panel**) and $\left(10\bar{1}1\right)$ surface (**bottom panel**) as computed by GGA + U; orderings of predicted directions of cobalt oxide growth are provided on the right. Below structures, pDOS in a.u. (range −2.5 to 2.5 states/eV) for 3*d* orbitals of each distinguishable Co surface atom and 2*p* orbitals of O atoms. Grey, dark blue, and light blue spheres represent Co (II) up spin, Co (II) down spin, and Co (III) atoms, respectively, with oxygen shown as red spheres.

Experimental studies of cobalt oxidation have observed the growth of cobalt oxides in the $\left(111\right)$ direction on the $\left(0001\right)$ surface of hcp Co [295]. The top panel of Figure 8 shows optimised magnetic ordering of the $\left(0001\right)$ surface with different oxygen coverages and the Co_3_O_4_ $\left(111\right)$ surface as modelled by the Hubbard-corrected generalised gradient approximation, GGA + U (U_eff_ = 3.0 eV) with accompanying densities of state (DOS). GGA + U is known to accurately capture the electronic and magnetic nature of metallic oxides and corresponding metal–oxygen structures, as also shown specifically for cobalt in the literature [296,297,298,299]. CoO is known to have antiferromagnetic ordering of type II (AF-II) as the most stable magnetic ordering below its Néel temperature (210 K) [300,301]. Co_3_O_4_ has magnetically active Co^2+^ ions located in the tetrahedral sites, while octahedral Co^3+^ ions do not have a permanent magnetic moment [302,303]. Bellow the Néel temperature (~40 K), the antiparallel magnetic ordering within the tetrahedral sublattice of Co_3_O_4_, due to the lack of magnetic carriers in the octahedral positions, yields a fairly low material magnetisation. Hence, oxidation at room temperature indicates a partial loss of magnetisation upon the formation of magnetically dead cobalt oxide layers on the surface.

Initial oxygen adsorption on the $\left(0001\right)$ surface, represented by low adsorbate coverages (0.33 ML), results in a detectable prolongation of Co–Co bonds between the oxygen-interacting and remaining Co atoms without changing the ferromagnetic nature of the cobalt surface. An increase in the magnetic moments of Co atoms that bind to oxygen (*d*_Co–O_ = 1.95 Å) is captured at 1.98 to 2.06 µ_B_, with 2.37 µ_B_ calculated for the elevated Co atom. This corresponds well to the experimental suggestions of Co–O formation with CoO character and an expanded intralayer lattice parameter [304], where the Co–O distance at the $\left(111\right)$ surface of CoO is approximately 1.85 Å and magnetic moments of Co atoms range from 2.30 to 2.60 µ_B_. For medium coverages (0.67 ML), oxygen atoms adsorbed such that three aligned surface Co atoms each formed bonds with three O atoms. Predicted magnetic coupling consists of Co atoms bonded to three O atoms which have spin down antiferromagnetic orientation and significantly reduced magnetic moments of 0.50–0.68 µ_B_, whilst the rest are oriented spin up and show values of 1.20–1.85 µ_B_. Finally, the optimised structure at full monolayer (1.00 ML) oxygen coverage, including bond lengths, DOS, magnetic moments, and magnetic orderings, implies the initialisation of continuous oxide formation on the $\left(0001\right)$ surface. The full first layer is predicted to have minimal magnetisation of 0.35 µ_B_ with ferromagnetic coupling. The main 2*p* O and 3*d* Co hybridisation peaks are situated between −0.5 and −2.0 eV, and between −4.0 and −5.0 eV, which is consistent with the nonmagnetic Co(III) ions of Co_3_O_4_. Changes in the magnetic moments of second-layer cobalt atoms are within 0.20 µ_B_ for any oxygen coverage.

Oxidation of the ($10\bar{1}1$) surface has not as yet been probed experimentally. The bottom panel of Figure 8 shows optimised magnetic ordering of the ($10\bar{1}1$) surface with different oxygen coverages as modelled by GGA+U (U_eff_ = 3.0 eV) plus accompanying DOS. For coverages of half a monolayer (0.50 ML) or less, no changes in the ferromagnetic coupling of surface cobalt atoms are observed and the calculated magnetic moments are in the range of 1.91–2.23 µ_B_. For higher coverages (0.75 ML), the results show changes in the magnetic coupling of certain surface atoms, with a further increase in the magnetic moments to 2.25–2.66 µ_B_. At full monolayer coverage (1.00 ML), half of the surface atoms had antiferromagnetic coupling and magnetic moments of 2.38–2.55 µ_B_, while the rest experienced ferromagnetic coupling with reduced magnetic moments at values of 1.88–2.20 µ_B_. Because of the integration of adsorbate atoms in the structurally arranged rows on the surface, deeper layer Co atoms also feel the presence of oxygen, causing enhancement of the magnetic moments to 1.85–1.95 µ_B_.

Reconstruction of the initial row-like geometry of the ($10\bar{1}1$) surface through the full monolayer oxygen adsorption indicates that the orientation of the cobalt oxide growth would more likely be through the CoO $\left(011\right)$ or CoO $\left(100\right)$ surfaces compared to the $\left(111\right)$ directional growth at the $\left(0001\right)$ surface. Experimentally suggested rearrangement of the channel-characterised $\left(11\bar{2}0\right)$ hcp Co surface towards the centred lattice of the CoO (100) plane [305] through movement of Co atoms and centring of the adsorbed oxygen between four Co atoms was successfully obtained within the same computational setup. Compared to the $\left(11\bar{2}0\right)$ surface, because of the prolonged Co–Co distances, further reconstruction towards the CoO $\left(100\right)$ faceting is not to be expected on the ($10\bar{1}1$) Co surface since already at 1.00 ML certain oxygen atoms were unable to form bonds with more than two surface atoms. Therefore, the $\left(011\right)$ surface was suggested as the orientation of CoO growth on the ($10\bar{1}1$) surface.

Decomposition of oxygen-induced magnetic effects on an atom-to-atom basis thus allows for precise insight into the nature of the oxidised surface layer, which can be used to predict the directional growth of oxide phases as well as further changes in the magnetic moments of slab layers. Similar to the layer progression of magnetic moments for bare slabs, oxygenated surfaces also suffer from the directional aspect of established interactions, where planar adsorption of oxygen cannot account for the morphology-governed adsorption on mNPs.

Following Van Vleck’s proposal [306] of spin–orbit coupling induced MCA, calculations of the magnetic anisotropy of metallic bulk phases and slabs have been carried out at different levels of approximation, pioneered by Brooks [307], Fletcher [308], and Daalderop [309,310], which have given satisfactory agreement with experimental methods. An interesting phenomenon of spin reorientation in metallic 2D systems has been observed for supported magnetic films (Fe/Ag(001) [311], Co/Au(111) [312], Ni/Cu(001) [313]). The crucial parameter that affects the direction of magnetisation is the film thickness, as it can induce change in the spontaneous magnetisation axis due to the competition between the interface anisotropy and the effective bulk anisotropy, which includes magneto-crystalline and shape anisotropy energies. It has been recently demonstrated that the electronic states can be responsible for the oscillations of the magnetic anisotropy in Fe(001) thin films with periods of five to nine monolayers since the quantum well states formed by *d* electrons change position in reciprocal space depending on the magnetisation direction [314,315,316]. Magnetisation-dependent spin–orbit gaps were also observed when the system symmetry was decreased compared to the bulk symmetry. Other factors influencing the interface or bulk terms include temperature conditions (annealing [317]), the presence of surfactants (capping layers [318,319]), and the stacking sequence [320], to name a few, and hence the spin reorientation in the metallic systems also depends on each of these parameters.

**Figure 9:**
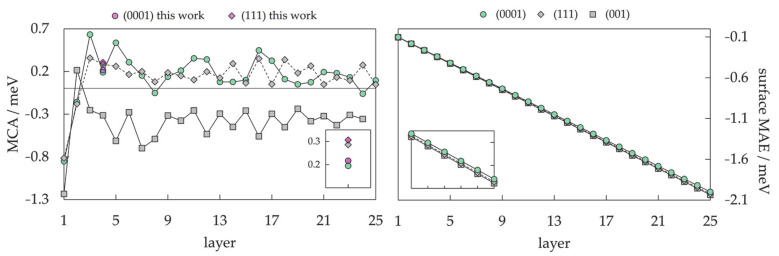
MCA energies for different fcc and hcp Co surfaces of varying thickness calculated at the LSDA level of theory (**left**) and estimated shape anisotropy (**right**). Symbols are the same for both graphs. Data are taken from Zhang (2009) [321]. Legends are the same for both graphs.

The thickness dependence of the MCA energy of cobalt films for different slab arrangements (hcp $\left(0001\right)$, fcc $\left(001\right)$, and fcc $\left(111\right)$, built from the experimental lattice constants) is shown on the left in Figure 9 at the local spin density approximation (LSDA) theory level [321]. In the progression from ultrathin to >10 Å slabs, significant oscillations are captured. For slabs with fewer than three atomic layers, interaction between the layers is more pronounced, resulting in a less systematic behaviour that deviates from the general trend. A certain degree of MCA energy convergence with respect to the slab thickness is also obtained as the number of layers increases, although the oscillations comparable to the total anisotropy values persist even for the thickest slabs considered. Cubic symmetry imposes a minimal barrier for the transition between any of the perpendicular magnetisation directions since its calculated anisotropy is less than a few μeV, meaning that the anisotropies of differently arranged fcc slabs are determined only by the in-plane lattice structure, considering that the bulk anisotropy is negligible. Thus, the converged values are actually the surface anisotropy energies, and their estimations are −0.38 meV for the $\left(001\right)$ fcc surface and 0.19 meV for the $\left(111\right)$ fcc surface. Note that a positive MCA energy means that the easy magnetisation axis is out-of-plane, whereas a negative MCA energy belongs to slabs with an in-plane easy magnetisation axis. The MCA energy of the hcp $\left(0001\right)$ slab shows a slightly negative slope, where the gradient of the linear fit for slabs with more than 10 layers is −0.015 meV. This is in good agreement with the MCA energy of the LSDA hcp bulk Co, which was calculated to be −0.010 meV. The value of the surface anisotropy for the hcp $\left(0001\right)$ surface has converged to 0.44 meV.

Another important contribution to the magnetic anisotropy of the system is the shape anisotropy energy. For 2D films, shape anisotropy can be estimated at 2μ_B_*M*_s_^2^, as shown for Co slabs in Figure 9 on the right. For both fcc and hcp Co slabs, the shape anisotropy energy has almost the same linear dependence with respect to the thickness.

Calculations based on the unrestricted GGA relaxation of the four-layer surface slab models were conducted for comparison, as shown in Figure 9 on the left, and agreement in the results is satisfactory. Predicted anisotropy energies for the $\left(0001\right)$, ($10\bar{1}1$), and $\left(111\right)$ Co slabs are hence 81.4 μJ/m^2^ or 6.57 μeV/atom, −7.98 μJ/m^2^ or −0.70 μeV/atom, and 199.01 μJ/m^2^ or 16.63 μeV/atom, respectively. These MCA values arise from the same computational setup that captured the correct order of magnitude for bulk MCA of hcp and fcc cobalt (calculated at 4.16 × 10^5^ J/m^3^ or 27.20 μeV/atom and −1.68 × 10^4^ J/m^3^ or −1.54 μeV/atom; experiment at 0 K 7–8 × 10^5^ J/m^3^ or 65 μeV/atom and −2.36 × 10^4^ J/m^3^ or −1.6 μeV/atom [322]). Results of other experimental and theoretical studies conducted on self-standing Co films also showed varying values depending on the film thickness. From the anisotropy modelling of close-packed $\left(111\right)$ ultrathin transition-metal films, the MAE obtained for the Co (111) film was −0.28 meV [323]. A Néel model of the Co $\left(0001\right)$ surface predicted an MAE of 84 μJ/m^2^ [324], whereas measurements on the 1000 Å thick Co film resulted in an in-plane anisotropy volume of −7.2 × 10^2^ J/m^3^ [325].

2D Co systems deposited on different substrates have received a lot of attention for their unique magnetic properties, both experimentally [320,326,327] and theoretically [328,329,330,331]. For a nonmagnetic supporting material, such as a gold surface, measured MAE values correspond well to those calculated for unsupported extended slab models—experimental measurements on the Au/Co interface gave values of 53–70 μJ/m^2^ [332]. Another study of the Co/Au(111) system showed 17 μeV/atom out-of-plane magnetic anisotropy [333]. Supported ultrathin Co films are characterised by much higher anisotropy values. When ultrathin films of cobalt atoms were deposited on Rh(111) and Pt(111) surfaces, the MAE values measured were −0.37 ± 0.05 and −0.29 meV per Co atom, respectively [334]. The combination of Co and Ir in a three-layer system gave rise to a similar anisotropy of 0.46 meV per atom, whereas only 0.02 meV MAE per atom was calculated for the Fe–Co–Ir system [335]. Furthermore, significant oscillations were observed in the interlayer exchange coupling for systems of varying numbers of Co and 4*d* transition-metal series layers, and the following quantitative trends of magnetic properties were formulated as a result: across the noble metals and Pd, a ferromagnetic interlayer coupling was found; for the post-noble metals and most 4*d* transition metals (with the exception of Mo), an antiferromagnetic interlayer coupling was obtained, meaning that the choice of the metal can influence the easy axis of magnetisation [336]. The interface magnetic anisotropy of the Co $\left(0001\right)$ film was shown to increase by 36 μJ/m^2^ upon Co surface oxidation, and follow-up DFT calculations correlated this change with a positive charge increase of 0.54e^−^ per oxidised Co atom [337]. However, the MAE of extended metal slab models is strictly dominated by the surface anisotropy factor, whereas the three-dimensional shape anisotropy that often affects both intensity and easy axis of MAE of mNPs cannot be accounted for through the 2D models.

#### 3.1.3. Nanoparticle Model

**Figure 10:**
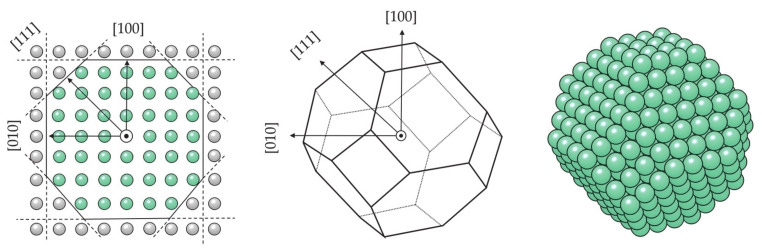
Illustration of the Wulff construction of an fcc particle. Growth direction of crystal facet planes is represented by black arrows, and the centre of the particle is represented by a black dot. Atoms enclosed in the equilibrium shape of the nanoparticle are coloured in teal, while the rest of the bulk atoms are grey. 3D morphology is shown in wire-frame and atomic representation.

A surface representation of mNPs deliberately neglects the role of undercoordinated particle sites, namely edges and vertices, and the effect of the full mNP geometric and electronic structure, which often differs from that of the bulk material. However, converged surface energies obtained by employing surface slab models can be implemented to build NP models using the well-known Wulff construction method [338,339,340]. The premise of the Wulff construction is that the length of a vector drawn normal to a crystal face from the particle centre is proportional to the surface energy of the corresponding facet, and the crystal equilibrium shape is obtained through the necessary minimisation of the total surface energy of the particle. In practice, there is an infinite number of possible surfaces that can be included in the model. Restraining the surface exploration to a set of those that are experimentally observed is a plausible strategy, whereas taking into consideration only the surfaces with low indices, which in general tend to include the most stable members, is a relatively reliable getaway because such surfaces are the most likely to be expressed in the morphology. Wulff NP construction of low-index facets has become a standard technique to build particle models, as shown in Figure 10. As the contributions from the particle edges are neglected in the Wulff construction method, the shape of small particles might deviate from the predictions, but experimental analysis indicates that particles in the range of a few nanometres are generally consistent with this construction principle [341,342].

**Figure 11:**
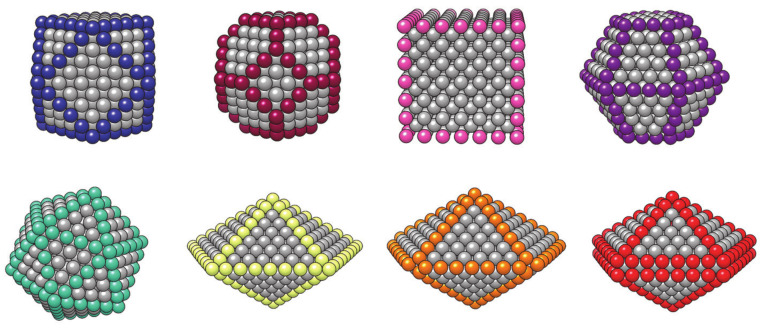
Models of crystalline NPs (left to right: fcc—cuboctahedron and truncated octahedron, bcc, and hcp) in top panel and noncrystalline NPs (left to right: icosahedron, regular, Marks, and Ino decahedron) in bottom panel.

More compact, quasispherical NP shapes can be constructed by applying symmetry operations over stable surface members to obtain the highest density of atoms within the NP volume, resulting in the multiple-twinned particle models. These are known as noncrystalline NP models, and the most common is the icosahedron composed of 20 tetrahedra with $\left(111\right)$ close-packed facets as their base and the central vertex as their apex, conjoined at 12 boundary vertices with a fivefold rotational axis, as initially proposed by Mackay [343]. The decahedron, on the other hand, is formed of five tetrahedra with $\left(111\right)$ close-packed facets, but its sphericity is far from that of the icosahedron, and it was first suggested by Ino [344]. Vertex and edge truncations, suggested by Marks and Ino [345,346], can be implemented to achieve a better surface-to-volume ratio. However, any noncrystalline motif is obtained at the expense of volume contributions for facets to be able to close the intersurface gaps. Some of the possible mNP models, both crystalline and noncrystalline, are represented in Figure 11.

Significant progress in the modelling of mNPs was made when moderately large NP models were implemented in DFT calculations for the simultaneous description of terrace sites of extended surfaces and low-coordinated edge and vertex sites. The NP model strategy is sufficient in providing the influence of the particle size and shape on the properties of interest by defining the so-called scalable regime where many properties (typically nonlocal—independent on the position of different atoms within the cluster, such as cohesive energy per atom) monotonously converge toward the bulk or extended surface limit. Other, more localised properties, which are related to the atoms of a particular region of the NP, such as adsorption energies of simple adsorbates, show invariance with respect to the number of atoms and slowly converge with the increase in the NP size.

**Figure 12:**
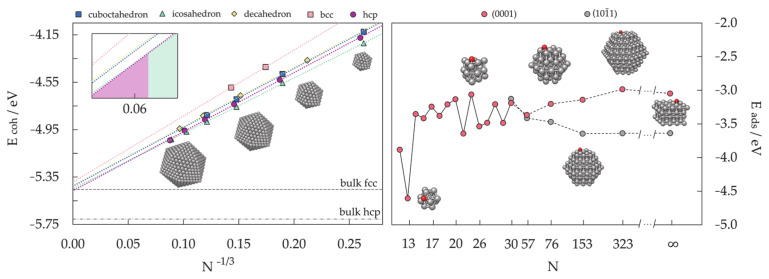
Linear fitting of the cohesive energies of Co NPs of varying morphologies with 55 < N < 1500 atoms with an insert capturing the crossover between the icosahedral and hexagonal shape (**left**) [239]. Progression of oxygen adsorption energies from nonscalable (2 < N < 50) to scalable regime of Co NPs with hexagonal symmetry (**right**); extended slab models are denoted as ∞.

Monotonous convergence of the cohesive energy of Co mNPs by applying the NP model is shown in Figure 12 (left) for various morphologies and particle sizes [239]. To limit the exhaustive number of calculations that would be needed if every NP size was considered, the choice of sizes of interest is usually driven by the well-known oft-recurrence of mNPs with a complete, regular outer geometry, designated as full-shell NPs. These numbers, known as magic numbers, are obtained through the mathematical relationships determined individually for each structural motif [347,348].

Size and morphology dependency of the energetic stability of a Co mNP is well represented by linear regression and can be extrapolated to very large mNP sizes. For sizes below 100 atoms, the stability of motifs based on the successive increase in the cohesive energy per atom decreases going from the most stable icosahedron, over fcc truncated octahedron, hcp, fcc cuboctahedron, and decahedron, to bcc as the least stable shape of Co mNPs. Differences in energies for the clusters with the same number of atoms between any two shapes are close to or less than 0.10 eV per atom. The icosahedron is identified as the most stable shape throughout the whole range of small and medium cluster sizes, consistent with the experimentally determined predominance of this noncrystalline shape within particles of up to N = 800 atoms [349,350]. The icosahedron-to-hcp transition is predicted to happen at around N ≈ 5500. An enlarged view of the intersection is represented as an insert in Figure 12 (left).

A linear fitting approach based on the magic-numbered mNPs and a subsequent determination of the stability trends is constrained by the assumption that the stability order is maintained for the remaining, nonmagic particle sizes. Based on the experimental indications of the simultaneous occurrence of more than a single geometrical shape at practically any size, linearly interpolated morphology transitions of magic-numbered clusters serve only as a guideline to the predominating geometry. A promising qualitative agreement was found in recent works that have conducted a full sampling of the energy landscape beyond the magic numbers, where the dominant mNP shape coincides with the one predicted by a ‘simple’ magic number linear fit [351,352,353]. The noncrystalline/crystalline distributions thus obtained for Co clusters with magic numbers of atoms represent a good reference point for defining crossover sizes between the structural motifs. Note that shape alternations could occur in reported stability windows, but they should nevertheless be expected to contain the highest proportion of the energetically most favourable structure.

An example of the convergence of more localised properties is the size-dependent progression of the adsorption energy. Calculations of the adsorption behaviour of simple adsorbates, but also of more complex molecules, have captured the nonscalable regime of the smallest metallic cluster models and convergence of adsorption energies for particles composed of approximately 50–60 atoms [273,354,355,356,357,358,359,360,361]. Size dependence of oxygen adsorption on small Co clusters with hexagonal symmetry and large crystalline hcp NP models, as well as on the extended slab models for the hcp $\left(0001\right)$ and $\left(10\bar{1}1\right)$ surfaces that appear in the hcp mNP morphologies, is depicted in Figure 12 (right) [362]. The 13-atom hcp Co cluster shows by far the most favoured oxygen adsorption, followed by notable oscillations in the adsorption strength established for succeeding cluster sizes. A nonscalable regime extends to the Co clusters with 30 < N < 50 atoms, similar to what was observed for other metallic systems. For particles with more than 30 atoms, distinctive facet areas can be assigned to the $\left(0001\right)$ and $\left(10\bar{1}1\right)$ hcp Co surfaces, with each showing unique adsorption behaviour based on their structural arrangements. Nevertheless, adsorption interaction on both constituent hcp mNP surfaces eventually converges to the respective extended slab adsorption energies. The difference in adsorption strengths on the large 323-atom hcp mNPs is insignificant compared to the periodic slab models.

The emergence of the scalable regime occurs at different cluster/NP sizes for distinct properties. The density of states, and, accordingly, the electronic structure, evolves rapidly towards bulk-like behaviour, which enables the use of relatively small mNP models for evaluating the chemical activity of sites characterised by low coordination numbers. The activity toward the adsorption of atomic or molecular species is often evaluated using either the d-band centre as the electronic descriptor or the generalised coordination number as the geometric descriptor, since it was found that the two are linearly correlated [363].

As a general convergence rule, properties are essentially considered converged when the local atomic environment is maintained with the further addition of atoms. For example, the adsorption strength is mostly conditioned by the adsorption site, followed to a substantially decreased extent by the influence of the first atomic neighbours and the second-shell neighbours, whereas neighbours of the third shell have negligible contributions. This approach can be extended to other local or regional properties.

To obtain an optimal description of the mNP properties of interest, it is crucial to choose the particle size that will reliably capture the effects of the studied morphology without accentuating either cluster-dominated behaviour or the monotonicity of the extended slab perspective. This is especially important when investigating the magnetic properties, because they are easily influenced by symmetry factors, directional interactions, and local atomic environment.

**Figure 13:**
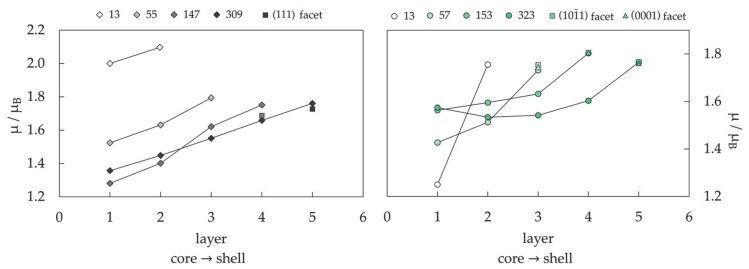
Magnetic moment per Co atom, µ, as a function of the Co NP layers for increasing nanoparticle size—**left**: icosahedral NPs, **right**: hcp NPs. Layers are numbered going from the core to the shell of the NP.

Figure 13 represents the core-to-shell progression of magnetic moments in differently sized icosahedral and hcp mNPs. The 13-atom cluster size from the nonscalable regime clearly stands out for both core and shell segments. For larger mNPs, core and inner-most layers show 15–25% lower magnetic moments compared to the surface itself or to the layers in close proximity to the surface. The increase in the magnetisation of surface atoms is hence 3 to 5 times more pronounced than in the case of extended slab models. Additionally, some distinctions in the overall core-to-shell trends have also been captured compared to the layer-to-layer progression of extended slab models.

**Figure 14:**
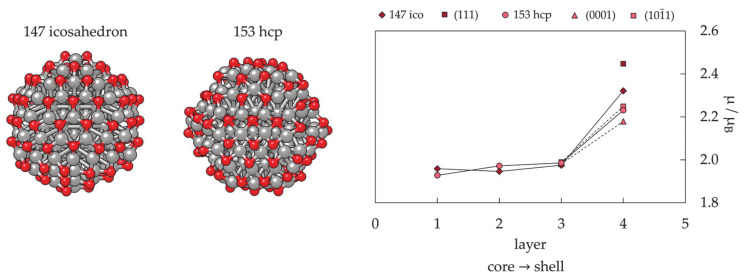
Structures of oxidised 147-atom icosahedron and 153-atom hcp Co mNPs (**left**) and corresponding layer-averaged magnetic moments per Co atom with core-to-surface progression, 1 being the core atom and 4 being the outer shell layer (**right**). Average magnetic moments of $\left(111\right)$, $\left(0001\right)$, and $\left(10\bar{1}1\right)$ facets are also given.

These differences become more pronounced when the effects of oxidation on the magnetic moments are examined for 147-atom icosahedron and 153-atom hcp Co mNP models (Figure 14). Compact organisation of Co atoms within the mNP morphologies allows for less flexible rearrangement to accommodate atomic adsorbates such as oxygen into the structure, in contrast to the frequently observed adsorption-induced reconstruction of the top-most layers of a surface slab [364,365,366,367,368,369,370]. For this reason, enhancement of the magnetic moments of the layer placed immediately below the vacuum-exposed outer shell obtained upon oxidation is significantly modified in the absence of surface rearrangement. The increase in the average magnetic moments for Co atoms within this layer is about 8% for both icosahedron and hcp Co mNPs, whereas corresponding slab enhancements for the $\left(111\right)$ and $\left(10\bar{1}1\right)$ surfaces are 30–40%. The increase in the surface shell magnetic moments is 11–20%. Additionally, elongation of the inner Co–Co distances upon the interaction of surface Co atoms with oxygen adsorbates lessened the volumetric strain and consequently the quenching of the magnetic moment of the central atom. This adsorbate-induced effect could not be captured within extended slab models.

**Figure 15:**
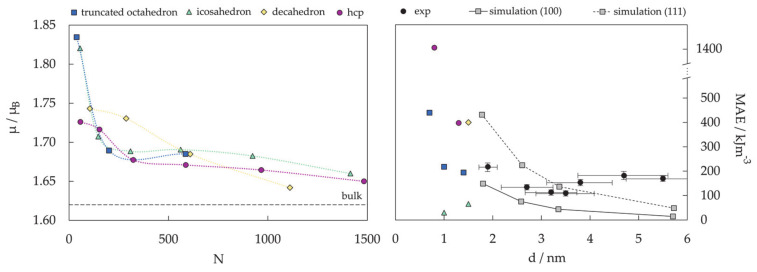
DFT-predicted progression of average magnetic moment per atom, µ, (**left**) and comparison between DFT-calculated, experimentally measured, and Néel pair surface correlated magnetic anisotropy energies, MAE, (**right**) with the increase in the size for Co mNPs of varying morphologies. DFT results are taken from Farkaš (2021) [384], and experimental and Néel pair modelling results are taken from Oyarzún (2005) [371].

The morphological dependence of the magnetic properties of Co mNPs is captured in Figure 15. Respectable shape deviation of magnetic moment per Co atom is observed only for the smallest mNPs, where each morphology has a unique share of vertex and edge sites. These differences wear off with the increase in the mNP size, when average magnetic moments start rapidly converging towards the bulk Co value. The MAE of mNPs, on the other hand, depends strongly on the shape anisotropy arising from the varying morphologies for all mNP sizes. This is a specific aspect of magnetic nanoparticles, and it is impossible to capture it within the extended slab models. The computational expense of noncollinear DFT calculations limited the determination of MAE to particles with 1–2 nm diameters (50–200 atoms), and the results obtained are shown in Figure 15 (right), together with the experimental data measured for embedded Co mNPs [371].

DFT results indicate that the Co mNPs with hexagonal symmetry have the highest MAE values of 1412.5 kJ/m^3^ for 0.8 nm and 397.9 kJ/m^3^ for 1.3 nm diameter mNPs. Calculated MAE for the 1.5 nm decahedron is 398.2 kJ/m^3^, whereas the other noncrystalline icosahedron shape is characterised by very low anisotropies, 30.2 and 65.6 kJ/m^3^ for 1.0 and 1.5 nm mNPs, respectively. MAEs of 0.7, 1.0, and 1.4 nm fcc truncated octahedra from DFT calculations are 439.9, 218.0, and 194.5 kJ/m^3^ and correspond well to the experimental anisotropy trend. Experimental data have been complemented by the Néel pair modelling to correlate the observed MAE features to the increased importance of the exposed facets, namely those of the (100) and (111) fcc Co surfaces, which is also shown in Figure 15 (right). The addition of a single facet does not contribute significantly to the modifications in the shape, but it is sufficient to break the symmetry and induce a change in the anisotropy of the entire particle. Such increase in the surface area successfully reproduced both experimentally observed effects, the increased MAE values with respect to the bulk for the smallest sizes and the varying trend in MAE with decreasing size. However, even though the addition of a single facet decreases the general enhancement in magnetic anisotropy for larger particles, it is nevertheless possible to obtain very large MAE values because of the shape-induced anisotropy. This means that notably polycrystallinity, which is expected in large particles due to the fabrication mechanism, plays an important role in reducing the contributions of crystal symmetry-breaking to the global anisotropy. Defects have, on the other hand, been proposed as responsible for uniaxial anisotropy in larger cobalt or iron mNPs (8–20 nm) [372,373].

Other experimentally measured MAE energies are reported in the range of 6.0–30.0 kJ/m^3^, consistently above the Co bulk values (2.7 kJ/m^3^ fcc and 4.4 kJ/m^3^ hcp) [374,375,376]. Geometrical shapes of the particles are not always provided in these studies, but it is suspected that they are mostly crystalline fcc or icosahedral Co mNPs. Moreover, several studies of various mNPs and for different sizes have observed the coexistence of crystallographic structures both in the gas phase and deposited [377,378,379,380], especially without further annealing. Comparable strong variations with values of the same order (between 10 and 400 kJ/m^3^) have been derived for icosahedral Co mNPs in the 3.1–4.3 nm size range [381]. As hexagonal structures in Co mNPs are expected to compete in stability only at larger sizes above 20 nm [239,382], crystalline structures of fcc features are naturally dominant in any size range up to this critical size, and the general trend obtained in the experiments cannot be specifically assigned to the exact crystalline structure. For a specific case of 3.0 nm truncated octahedral particles, experimentally derived anisotropy constants are in the range of 10–200 kJ/m^3^ [383].

A combination of DFT simulations and NP models is hence a very powerful tool in predicting the magnetic behaviour of mNPs, and it corresponds very well to the complexity of the size- and morphology-dependence problem. With improvements in computational power and performance, it has become possible to utilise the advantages of the NP models over couple-atom clusters and extended slabs and to capture reliable values of magnetic moments, anisotropy energies, and adsorbate-induced alternations in the magnetic behaviour of mNPs [384]. These advances are expected to facilitate the research on mNPs with favourable properties for MRI contrast and magnetic nanoparticle hyperthermia agents.

### 3.2. Protected mNPs: Ligand Effects on Magnetic Properties

Two main features dominate the magnetic properties of mNPs: (1) their physical appearance, i.e., size and morphology, and (2) surface effects, i.e., symmetry breaking of the crystal structure at the mNP surface, canted spins of surface atoms, surface oxidation, or chemical effects induced by the bonding of surfactant molecules.

Since gas-phase mNPs have limited stability and can only exist on the order of a few milliseconds, their use in new technologies is based on more practical systems whose longevity is provided through the stabilisation by surface functionalisation [385,386,387]. Functionalisation molecules also passivate the mNP by forming bonds with the reactive surface metal atoms directly exposed to the environment. Unfortunately, the attachment of surfactants is notoriously correlated with the quenching of mNP magnetic moments [388,389,390,391]. In general, the extent of ligand-induced changes in magnetic behaviour depends on the metal–ligand pair in question. The origin of magnetic moment quenching of respective metal atoms in metal–ligand complexes has been linked to two electronic parameters: changes in the *d*-electron count of the metal and changes in the energy level splitting of the metal *d* orbitals through a ligand field effect [392,393,394]. These ligand-induced changes are indications of the possibly strong ligand effects on magnetic properties of mNPs where ligand-induced changes could scale as a function of the mNP size.

Ligand effects have been recognised as important factors in the implementation of mNPs in biomedicine, since functionalisation often comes at a cost of not only reduced magnetisation, but also changed surface anisotropy. It has been recently demonstrated that the deposition of a self-assembled monolayer of alkanethiolates on an ultrathin Co film grown on Au(111) induces a spin reorientation transition from in-plane to out-of-plane magnetisation, changing the anisotropy values [333]. Computer simulations of these issues have often relied on the representation of mNPs as extended slab models because of the cost of noncollinear calculations. However, this approach causes even more uncertainties when estimating ligand-induced changes in magnetisation than in the simple assessment of the adsorption reactivity, because the neighbouring shells of atoms play a minimal role in the adsorption strength, while their contribution to the global magnetic behaviour is much more prominent.

To make a connection between the impact of ligands on the emergent properties of coordination complexes and in larger ligand-functionalised metallic structures, systematic studies of a combination of varying Co mNP size and a series of ligand shell compositions have been conducted.

**Figure 16:**
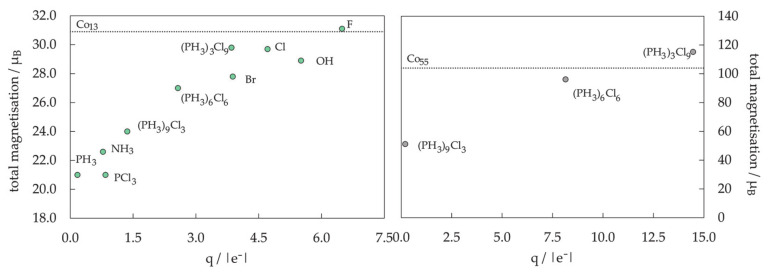
Total magnetic moment as a function of Bader charge for varying ligand shell composition on 13-atom (**left**) and 55-atom (**right**) icosahedral Co mNPs. Dotted lines indicate values of the bare mNPs. Data are taken from Hartmann (2016) [395].

Figure 16 shows the linear-like relationship between the total magnetic moment and the Bader charge on the Co core for different ligand shells and two core sizes, a 13-atom Co cluster and a 55-atom Co mNP. In general, there is a net quenching of the total magnetic moment relative to the bare cluster for each system, due to spins being paired in surface–ligand bonds. However, as the charge on the core becomes more positive, the total magnetisation increases. The intensity of the quenching is preserved with the increase in the particle size [395].

The average local magnetic moments of each Co atom were found to span a wide range of values as a function of the ligand shell composition [396]. The 13-atom cluster-PH_3_ ligand system quenches magnetic moments of all Co atoms from the bulk value of 1.72 to 1.61 µ_B_, whereas a fluorine ligand shell results in an increased atomic Co magnetic moment of 2.39 µ_B_. The 55-atom mNP–PH_3_ analogue experiences similar local magnetic moment quenching, even at the mNP centre. The 55-atom mNP–chlorine system also follows the trends of the chlorine-functionalised 13-atom cluster, with the chlorine ligands localising charge and increasing the magnetic moments of surface Co atoms. Analysis of bond lengths and surface–ligand bond angles showed that these alternations in atomic magnetic moments are not driven by geometry modifications due to functionalisation, but depend only on the type of ligand. This provides continuity in the locally induced magnetisation changes over differently sized metallic cores.

**Figure 17:**
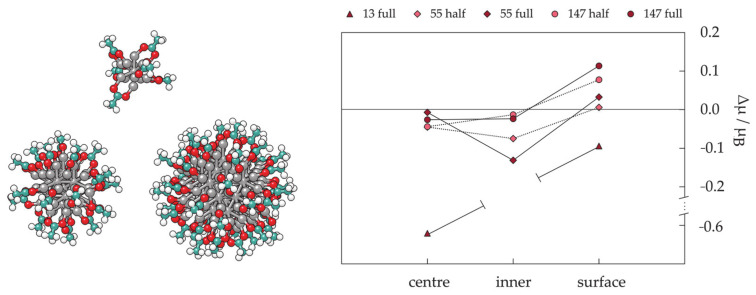
Ligand-induced changes in the magnetic moments of centre, inner, and surface segments of the 13-, 55-, and 147-atom icosahedron Co mNPs functionalised by a half and full acetic acid coating. Δµ is the difference between the average magnetic moment of each segment in unprotected and protected mNPs.

The transferability between the core size and magnetic moments of protected Co mNPs was confirmed through investigations of biomedically relevant ligands, namely carboxylic acids. Considering the scaling of ligand-induced changes in the magnetisation as a function of the Co NP size, half and full monolayer coverages of acetic acid have been modelled by DFT with the aim of identifying general trends. Progression of ligand-induced changes in the magnetic moments within the centre, inner, and surface segments of icosahedral Co mNPs with varying sizes (N = 13, 55, 147) upon carboxylic acid functionalisation is shown in Figure 17. Changes in the magnetisation of 55- and 147-atom Co mNPs with the increase in the density of the ligand shell correspond very well for each segment of the two mNP sizes, indicating that the smaller NP model is sufficient to capture the magnetic behaviour of protected Co mNPs.

**Figure 18:**
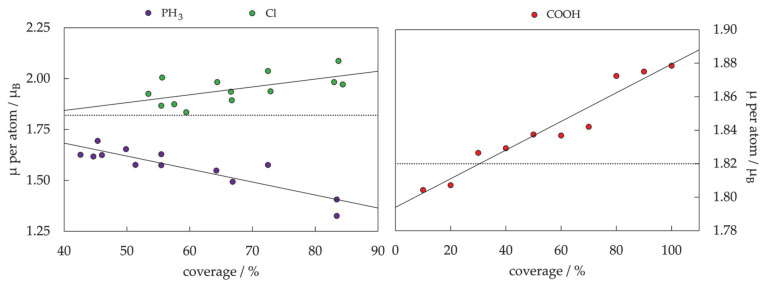
Average magnetic moment per Co atom, µ, as a function of ligand coverage for icosahedron Co mNPs passivated with Cl and PH_3_ (**left**) and COOH (**right**). Average magnetic moment per Co atom for unpassivated 55-atom Co icosahedron is added as the dotted line. Data on the left are taken from Hartmann (2018) [396], and data on the right are taken from Farkaš (2021) [384].

To gain more detailed insight into the coverage-dependent changes in the magnetic properties, average magnetic moments per Co atom are given as a function of the percent ligand coverage for Cl-, PH_3_-, and COOH-protected Co mNPs in Figure 18. In the case of each ligand type, there is a strong correlation between ligand coverage and resulting magnetic moment per Co atom, yielding a linear trend as was the case for the total magnetic moments as a function of the core charge. However, different ligand binding types can lead to both an increase and a decrease in the average magnetic moment of varying intensities compared to the moments of the unprotected mNPs. Higher Cl coverage has an enhancing effect on the atomic magnetic moments, where the line of best fit indicates a 0.0043 µ_B_ increase per percent coverage. However, an increasing magnetic moment arising from the passivating shell composed of acetic acid molecules shows a significantly slower growth of 0.0009 µ_B_ per percent coverage. In contrast, a PH_3_ shell leads to the quenching of the average atomic magnetic moment by 0.0065 µ_B_ per percent coverage. Taken together, the deviations in average atomic magnetic moment as a function of ligand identity and coverage are evidence that the composition and density of the passivating shell have a stronger impact on magnetisation than the size of the Co core.

**Figure 19:**
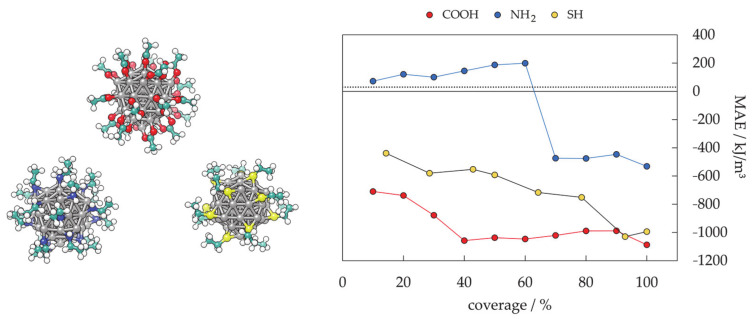
Structures (**left**) and magnetic anisotropy energy, MAE, as a function of ligand coverage (**right**) of COOH-, NH_2_-, and SH-passivated 55-atom icosahedron Co mNPs. MAE of unpassivated 55-atom Co icosahedron is added as the dotted line. Data are taken from Farkaš (2021) [384].

To capture ligand effects on the mNP anisotropy, ligand-protected icosahedron Co mNPs with varying coverages of biomedically relevant ligands (carboxylic acid, thiol, amine) were modelled. The calculated anisotropy energies are shown as a function of coverage in Figure 19. Starting from a very low value of 30.2 kJ/m^3^ for the unprotected 55-atom Co icosahedron, all three ligand families enhance the MAE with an increase in the coverage density. The rates of this increase are, however, ligand-dependent. Amine passivation resulted in the lowest enhancement of MAE, calculated for a 100% coverage at 531.4 kJ/m^3^. A change in the direction of the easy axis of magnetisation was also captured for amine-protected mNPs with coverage densities between 60 and 70%. There is a steady increase in the MAE of acid- and thiol-passivated Co mNPs as the protective coatings become denser, with acidic ligands showing a slightly higher enhancement rate per percent coverage. However, for the maximum coverages of 90 and 100%, MAEs of both acid- and thiol-protected Co mNPs reach similar energies with values between 987.0 and 1087.0 kJ/m^3^.

Overall, ligand binding induces local changes on the atoms of Co cluster and mNP systems, analogous to the effects in the ligand field theory. However, because the local coordination site is a component of a larger nanocluster, the impact of each ligand is observed beyond the metal centre to which it is directly bound. Furthermore, the chemical identity of the ligand within each binding motif provides a fine-tuning mechanism on the exhibited magnetic behaviour through the connection between the electronegativity of the functional group and the amount of electron density withdrawn from the core. Finally, as the size of the mNP increases, ligand-dependent magnetic properties persist, although they are slightly dampened.

### 3.3. Alloyed mNPs: Effects of Interfaces on Magnetic Properties

The applicability of single-component mNPs is restricted by limited property-tuning possibilities. To overcome this limitation, mNPs can be modified through the construction of bimetallic architectures consisting of two distinct metals, one or both of which should be magnetic [397,398,399,400,401,402]. Bimetallic (and multimetallic) mNPs, often referred to as magnetic nanoalloys, present properties with a very high degree of tunability owing to the variety of morphologies they can adopt. Their morphology is specified not only by the geometric structure, as in the case of the monometallic systems, but also by the chemical ordering of its components, which corresponds to the arrangement of the two metallic phases within the specified geometry.

The expectations of improved magnetic properties of monometallic mNPs by incorporating additional metallic phases originated from the very large magneto-crystalline anisotropy of the respective bulk and thin-film alloyed materials [403,404,405,406,407,408]. DFT calculations were shown to be capable of quantitatively describing the MAE and the orbital magnetisation in these alloys [409,410,411,412]. For example, in the case of the L1_0_ CoPt crystal, DFT predicted an MAE of 4290 kJ/m^3^ [227], very close to the experimental value of 4000 kJ/m^3^ measured by Eurin [413] and much higher than that of the fcc or even hcp Co bulk (23.6 and 700–800 kJ/m^3^).

Nevertheless, it was observed that the magnetic properties of alloyed mNPs could become worse than those of bulk metals, in contrast to the monometallic mNPs. The magnetic anisotropy energy of L1_0_ CoPt NPs was found to be 385 kJ/m^3^ [414] or 1700 kJ/m^3^ [415], both values much smaller than that measured for the bulk L1_0_ CoPt crystal. Moreover, a clear reduction in the saturation magnetisation of CoPt mNPs was captured with a decrease in the particle size [416]. Bimetallic mNPs are still promising for many applications because, despite the deterioration in the magnetic behaviour with respect to bulk alloys, which was suggested to be caused by surface effects (surface adsorption and surface spin canting [417]), their anisotropies and magnetic moments substantially exceed those of the monometallic counterparts. For example, distinct L1_0_ orderings in CoPt mNP systems have already shown how improved MAE can translate into superior catalytic performance in fuel cells [418].

The central difficulty in predicting magnetic properties of bimetallic mNPs lies in the complexity of the possible combinations in composition, geometry, and chemical ordering. Compared to the intermixed L1_0_ state of CoPt NPs, surface-segregated cuboidal counterparts have shown magnetic moments reduced by 0.52 µ_B_ and a 19% decrease in MAE. More pronounced, when the cuboctahedral morphology was considered, reductions in the total magnetic moment and CoPt mNP anisotropy were of the order of 4.96 µ_B_ and 45%, respectively [227].

Finding the optimal bimetallic mNP morphology for a specific composition of two metallic phases by employing MD simulations has shown good agreement with proposed structures of experimentally synthesised systems [419,420,421,422,423,424,425]. Owing to the difficulties in assigning magnetic properties to a specific mNP morphology through experimental techniques, DFT simulations are also valuable in predicting the magnetic behaviour of bimetallic mNPs of varying geometries and chemical orderings.

As to their thermodynamic stability, the cuboctahedral shape with L1_0_ crystal order was obtained as the most stable geometric structure for large CoPt NPs, based on the DFT-calculated surface energies and Wulff construction theorem [426]. Specifically for CoPt mNPs with diameters below 2.5 nm, DFT calculations have directly predicted the multiply twinned icosahedral and decahedral mNPs to be more stable than the L1_0_ cuboctahedron [238].

For biomedical applications, the alloying of cobalt with more inert metals such as silver or gold has proved to be a valuable strategy to obtain biocompatibility and reduce oxidation. In accordance with the large differences in atomic radii in favour of inert metals, and weak Ag–Co/Au–Co miscibility predicted below 400 °C across all compositional space [427], theoretical studies have reported structures that favour Ag/Au surface segregation and the formation of core–shell orderings [402,428,429]. Experimental synthesis has confirmed theoretical predictions, and core–shell AuCo mNPs were found to have mostly icosahedral structure, although a novel morphology has been recently described consisting of a Co icosahedron surrounded by fcc Au facets [430,431,432]. In addition, reports have indicated that optical and magnetic properties may both be tuned by tailoring the size of the core and shell of Ag/AuCo mNPs. However, the fact that the surface of the nanoalloy is expected to contain mostly Au (or other noble and inert metals such as Ag or Pt) atoms does not invariably determine the chemical ordering of such systems. The size, structure, and chemical arrangement of bimetallic mNPs can be controlled experimentally throughout the synthesis protocol, allowing metal species to intermix and maximise the synergistic benefits available through their unique combination. For example, physical methods have been reported for the synthesis of AgNi disordered mNPs, which exhibited substantial enhancement in their optical limiting efficiency [433]. Core–shell AuNi NPs with inverted segregation (Au cores covered by Ni) have shown huge magnetisation, which was maintained even after the formation of NiO on the surface [434]. The hybrid AuCo systems are consequently also expected to offer unique properties in different chemical orderings because of the possible magnetoresistance effect and optical–magnetic bifunctionalities [435]. Although the limited literature on AuCo systems is almost exclusively focused on the core–shell structures, Marbella et al. have demonstrated the synthesis of discrete, composition-tuneable alloyed AuCo NPs with random ordering of Au and Co atoms, whose magnetic susceptibility can be tailored while maintaining almost identical particle size and surface chemistry [54]. Finally, a theoretical study has shown that magnetic effects can destabilise core–shell arrangement of AgCo and AuCo systems whose icosahedral structures remain preferential, but peculiar quantum effects reverse the energetics in favour of intermediate compositions, presenting a much more thorough intermixing with cobalt atoms [33].

The magnetic anisotropy of AuCo mNPs was calculated to be an order of magnitude higher for L1_0_ ordering compared to the core–shell NPs of 1.5–2.0 nm in diameter. Predicted MAE values for cuboctahedron, icosahedron, and decahedron with the L1_0_ ordering were 928.3, 1038.3, and 1011.7 kJ/m^3^, respectively. A corresponding core–shell icosahedron showed MAE of 239.9 kJ/m^3^, while the MAE of a decahedron with core–shell structure was found to be 278.2 kJ/m^3^. Hence, L1_0_ bimetallic AuCo mNPs show a 15-fold improvement from the anisotropy of a 1.5 nm monometallic Co icosahedron, which will hopefully trigger improved efforts in the synthesis of these systems and their use in biomedical applications.

## 4. Conclusions

With the advances in computational power and continuous improvements of DFT methods, the implementation of explicit NP models in simulations of nanoparticle systems should be encouraged, since they are a more realistic representation and able to capture the property dependence on size and morphology. Whilst cluster and extended slab models can often offer valuable insight into the nature of metal–adsorbate interaction, they fail to capture the progression and directional effects of magnetic properties.

DFT simulations of NP models have shown excellent correspondence with experimental measurements of magnetic moments and magnetic anisotropy energies of cobalt mNPs. They are able to properly describe morphology-induced alternations in the magnetic features, calculate ligand effects on both the binding metal centre and the particle as a whole, and predict the outcomes of alloy interfaces of different chemical orderings. It is thus expected that *ab initio* methods can also provide accurate structures and properties of magnetic mono- and bimetallic NPs that do not necessarily have cobalt in their composition.

Incorporation of NP models in research efforts to optimise the magnetic behaviour of metallic magnetic nanosystems for biomedical applications could hence facilitate the smarter design of application-specific properties.

## Data Availability

Data sharing is not applicable for this article.
